# Hyaluronic Acid-Functionalized Highly Porous Polymeric Materials for Stem Cell Culture

**DOI:** 10.1021/acs.chemmater.5c00068

**Published:** 2025-07-22

**Authors:** Joshua D. Swindell, Despina Coursari, Charlotte E. Severn, Evelyn Calderon-Espinosa, Israa A. El-Shawaf, David M. Haddleton, Ashley M. Toye, Ahmed M. Eissa

**Affiliations:** † Department of Chemistry, 2707 University of Warwick, Coventry CV4 7AL, U.K.; ‡ School of Biochemistry, Biomedical Sciences Building, University Walk, Bristol BS8 1TD, U.K.; § Faculty of Engineering, 68791 Ain Shams University, Elsarayat Street 1, Abbaseya, Cairo 11517, Egypt; ∥ Department of Polymers, Chemical Industries Research Division, National Research Centre, El Bohouth Street 33, Dokki, Giza, 12622 Cairo, Egypt; ⊥ School of Pharmacy & Life Sciences and Research Institute of Healthcare Sciences, Faculty of Science and Engineering, 8695 University of Wolverhampton, Wolverhampton WV1 1LY, U.K.

## Abstract

We describe the synthesis of a functional macroporous polymer material and its potential use as a scaffold to support the 3D culture of hematopoietic stem and progenitor cells (HSPCs). Glycidyl methacrylate (GMA)-based emulsion-templated porous polymers (known as polyHIPEs) were prepared by photopolymerization and subsequently surface functionalized with hyaluronic acid (HA) using Huisgen azide–alkyne cycloaddition click reaction, inferring a high degree of functionalization based on the near-quantitative nature of the reactions. Quantitative azidation of GMA-based polyHIPEs is achieved by the ring opening reaction of epoxide rings with sodium azide. Reductive amination reaction is used to end-cap HA with alkyne functionality to be later clicked onto the azidified polyHIPE surfaces. The synthesized polyHIPE materials are characterized by ^1^H nuclear magnetic resonance (NMR), Fourier-transform infrared (FT-IR), Raman and X-ray photoelectron spectroscopy (XPS). Scanning electron microscopy (SEM) and compression testing are also conducted on the polyHIPE scaffolds to evaluate their morphological and mechanical characteristics, respectively. Biocompatibility and cell viability were assessed, along with preliminary stem cell culture experiments to evaluate the suitability of HA-functionalized polyHIPE scaffolds for stem cell maintenance and proliferation. These experiments revealed a notable increase of approximately 20% in CD36^+^ cell proliferation on HA-polyHIPE scaffolds compared to the control GMA-polyHIPE scaffolds. The multifunctionality served by HA, such as its biocompatibility, biodegradability, nonimmunogenicity, anti-inflammatory properties, antiangiogenic characteristics and binding ability to biological targets critical to mimicking stem cell environment, significantly advances the development of biological scaffolds for tissue engineering and regeneration.

## Graphical abstract

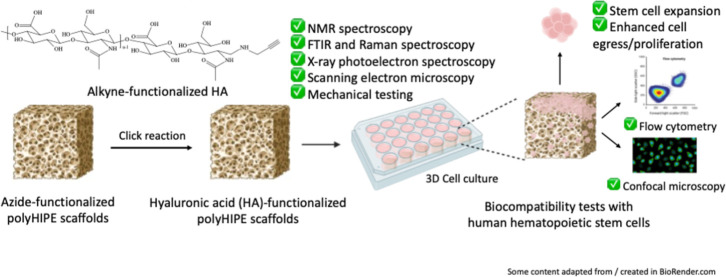

## 1 Introduction

In vivo, cells reside in a complex microenvironment known as the extracellular matrix (ECM) that provides biophysical and biochemical cues that direct cell growth and fate. Over the last few decades, there has been an increasing research focus on the development of synthetic scaffolds that recapitulate the specific properties of in vivo environments and ultimately emulate the cell ECM. Beside the architectural properties of scaffolds such as porosity and mechanical strength, concentration and distribution of surface functionalities including biomolecules (growth factors and binding ligands) are essential requirements to support cell growth, proliferation and function in vitro. ECM features influence stem cell differentiation, tissue regeneration, proliferation rates, migratory potential, and even disease states such as cancer.

One class of scaffolds that have recently been shown to provide support for tissue and matrix regeneration is macroporous polymerized high internal phase emulsion (polyHIPE) scaffolds, thanks to their high porosity, tunability, superior surface area, surface chemistry and robust mechanical properties.^1,2^ The application of polyHIPE scaffolds in supporting the 3D culture and tissue engineering of mammalian cells has steadily grown in recent years.^3^ A variety of polyHIPE biological scaffolds have been developed including polyurethane,^4^ thiol–acrylates,^5^ *N*-carboxyanhydrides^6^ and ε-caprolactone (PCL)-based structures^7,8^ that have shown their ability to provide good viability, migration, and proliferation of cells. Different cell types have been successfully cultured on polyHIPE scaffolds including mammalian hepatocytes,^9^ endometrial,^10^ pluripotent,^11^ and hematopoietic stem cells.^12^ However, the main limitations for the widespread adoption of polyHIPE scaffolds made by water-in-oil (W/O) emulsions in biomedical applications including tissue engineering are their inherent hydrophobicity and limited surface functionality. Efforts have been made to develop methodologies to introduce chemical functionalities onto polyHIPE surfaces including incorporation of functional monomers into the HIPE and postpolymerization chemical modification which have led to a steady increase in polyHIPE applications.^3,13,14^

Direct incorporation of functional comonomers became more feasible with acrylate and methacrylate-based polyHIPEs.^15^ For instance, inclusion of the reactive monomer, 4-vinylbenzene chloride, has been found to lower interfacial tension, reduce the void size, and later facilitate functionalization with amines.^16–20^ Nonetheless, the range of reactive comonomers that can be used is limited by their solubility, which could potentially lead to HIPE destabilization and changes to the polyHIPE morphology.^21^ Some reactive comonomers have been incorporated into polyHIPE material to present reactive surface groups for grafting.^22,23^ Over the past decade, postpolymerization surface functionalization has become an increasingly prevalent strategy to overcome the limitations associated with functional monomer incorporation.

This approach encompasses various techniques, including grafting “from” surface initiator groups, commonly used for further surface polymerization via halides or unreacted vinyl groups.^17,24–29^ Surface polymerization methods such as atom transfer radical polymerization (ATRP) and reversible addition–fragmentation chain-transfer (RAFT) allow for the homogeneous addition of functionalities with high surface coverage without compromising HIPE stability.^21,30^ Furthermore, alternative surface functionalization methods beyond these categories have been explored, including the entanglement of functional polymerizable amphiphilic surfactants and the use of “reverse” oil-in-water (O/W) polyHIPEs. Functional surfactants demonstrated a “one-pot” functionalization approach, eliminating the need for complex multistep procedures.^31,32^ A seemingly straightforward solution to address surface hydrophilicity involves employing reverse O/W polyHIPEs where the external continuous phase is water-based, making them more suitable for biological systems. Hydrophilic O/W polyHIPEs have seen gradual progress due to their inherent biocompatibility and biodegradability.^33–35^ However, these systems still face significant mechanical, thermal, and stability challenges requiring further optimization.^36^ Additionally, the internal droplet phase in O/W HIPEs requires a large volume of mostly toxic organic solvent which is more difficult to remove postpolymerization compared to an aqueous internal phase.

Another significant postpolymerization strategy involves grafting “onto” existing surface functional groups through chemical reactions to covalently attach desired functionalities. One method to introduce functionalities like amides and acid chlorides onto polyHIPE surfaces involves reacting surface esters.^37–40^ Another recent grafting “onto” approach utilizes thiol–ene acrylate systems that generate reactive, residual surface thiols for subsequent grafting of functionalities and biomolecules such as maleimide and acrylates.^11,12,41^ Moreover, grafting “onto” surface epoxide groups from the reactive comonomer glycidyl methacrylate (GMA) has been investigated for the direct attachment of biomolecules to enhance biocompatibility. While early GMA-based polyHIPEs exhibited significant void size variation, subsequent studies have addressed this issue and successfully grafted proteinase K, heparin, 6-aminocaproic acid, and various simple and multifunctional amines onto the surface via nucleophilic ring-opening reactions.^22,42–44^ Finally, “click” reactions^45^ have been employed to functionalize polyHIPE surfaces as a means of grafting “onto” approach. Thermal and UV-initiated radical-mediated thiol–acrylate^41^ as well as copper-mediated azide–alkyne cycloaddition (CuAAC)^45–48^ click reactions have been reported to produce well-defined functional polyHIPE materials. While most applications to date have focused on nonbiological, nonaqueous systems, the application of CuAAC click chemistry in a biological context could offer a facile, biologically compatible method for surface functionalizing polyHIPE scaffolds.

Efforts have been made to develop bioactive polyHIPE scaffolds for improved in vitro and in vivo applications.^9,11^ One bioactive macromolecule that has attracted much attention in recent years due to its role in many biomedical applications is hyaluronic acid (HA) or hyaluronan. HA is a naturally occurring, linear polysaccharide part of the glycosaminoglycans (GAGs) family. The chemical structure of HA comprises of a repeating disaccharide structure of d-glucuronic acid and *N*-acetylglucosamine units, linked by β(1 → 3) linkages.^49,50^ HA, found throughout the human body, plays an important role in the control of tissue hydration and the regulation of cell division, water transport, migration, differentiation, and regeneration.^51^ HA is also an essential component in the extracellular matrix (ECM) mediating cellular signaling, wound repair, morphogenesis, and matrix organization and could have a critical role in supporting synthetic immitatons.^52,53^

Importantly for potential cell culture and tissue engineering applications, HA binds to several widely found cell surface receptors such as CD44,^54–56^ CD168 (RHAMM),^56^ TSG-6,^57^ LYVE-1,^58^ and others, to influence cell behavior.^59^ HA-CD44 and HA-RHAMM interactions have attracted the most attention due to their reported role in metastasis and tumor growth.^51,54^ As a result, HA has already been incorporated into a very wide range of biomaterials to date from hydrogels to nanoparticles^60–63^ where several processing techniques have been employed including phase separation, rapid prototyping, freeze-drying and micropatterning.^49^ HA is then commonly incorporated by cross-linking and covalent modification.^64^ For instance, HA hydrogels have been extensively explored for 3D cell culture and tissue engineering, demonstrating their ability to support cell encapsulation, proliferation, and differentiation.^65,66^ To our best of our knowledge, despite the widespread use of HA in hydrogel-based scaffolds, the development of HA as a surface functionalization on macroporous polyHIPE scaffolds for enhanced cell culture applications remains largely unexplored.^67^ The development of HA-based scaffolds holds significant potential for advanced applications in biomedical materials.

We have previously demonstrated that polyHIPE scaffolds, inspired by human bone architecture, support hematopoietic stem and progenitor cell (HSPC) culture with long-term CD34^+^ cell maintenance and erythroid progenitor release, enabling large-scale reticulocyte production.^12^ Herein, we describe a method for surface functionalizing polyHIPE scaffolds with HA using the CuAAC click reaction. Our approach provides a platform for enhanced cell culture applications. Specifically, this study seeks to answer whether surface functionalization of polyHIPE scaffolds with HA, using this CuAAC method, enhances their capacity to support the growth and expansion of CD34^+^ cells. Initially, GMA-based polyHIPEs were synthesized by photopolymerization, then characterized by FT-IR spectroscopy, SEM, and mechanical testing. The GMA-polyHIPE scaffolds were then functionalized with azide groups as characterized by FT-IR and XPS. On the other hand, HA was functionalized by reductive amination with a terminal alkyne group and fully characterized by NMR, Raman, XPS and FTIR spectroscopy. To further demonstrate the successful functionalization with a terminal alkyne functionality, HA was attached to small model molecules using the CuAAC click reaction. Finally, HA was clicked onto the azidified polyHIPE scaffolds and these final materials were characterized by FT-IR, XPS and SEM. Preliminary cell culture experiments were performed to investigate the biological compatibility of HA-functionalized polyHIPE scaffolds, in terms of supporting growth and expansion of CD34^+^ cells.

## 2 Results and Discussion

### 2.1 Synthesis of GMA- and Azide-Based polyHIPE Materials

Different GMA concentrations were explored during HIPE preparation. An initial concentration of 10% v/v GMA resulted in low surface functionality (Figure S1, Supporting Information). Higher concentrations than 25% v/v led to phase separation, negatively affecting porosity as previously reported.^68^ Therefore, all subsequent experiments utilized 25% v/v GMA functionalized polyHIPE materials, referred to as GMA-polyHIPE.

GMA-polyHIPE materials were synthesized with a nominal porosity of ca. 90% as determined by water-to-oil phase ratios. SEM micrographs (Figure 1) revealed a highly porous material with fully interconnected open-cell morphology where the average void diameter was found to be 13.3 ± 3.7 μm which is deemed to be sufficient for most cell types infiltrate the scaffold. This interconnected porosity with suitable pore size is crucial for facilitating stem cell infiltration and nutrient diffusion in 3D culture. The open-cell structure is indeed a key requirement for the application of polyHIPE materials as scaffolds in 3D cell culture as it allows efficient diffusion of nutrition and waste to and from the cells residing in the scaffold. Upon azidification reaction, the morphology of the produced N_3_-polyHIPE material remained unchanged, showing the same interconnected porosity in the original GMA-polyHIPE material with an average void diameter within the error of the original material at 12.9 ± 3.3 μm (Figure 2).

**Figure 1:**
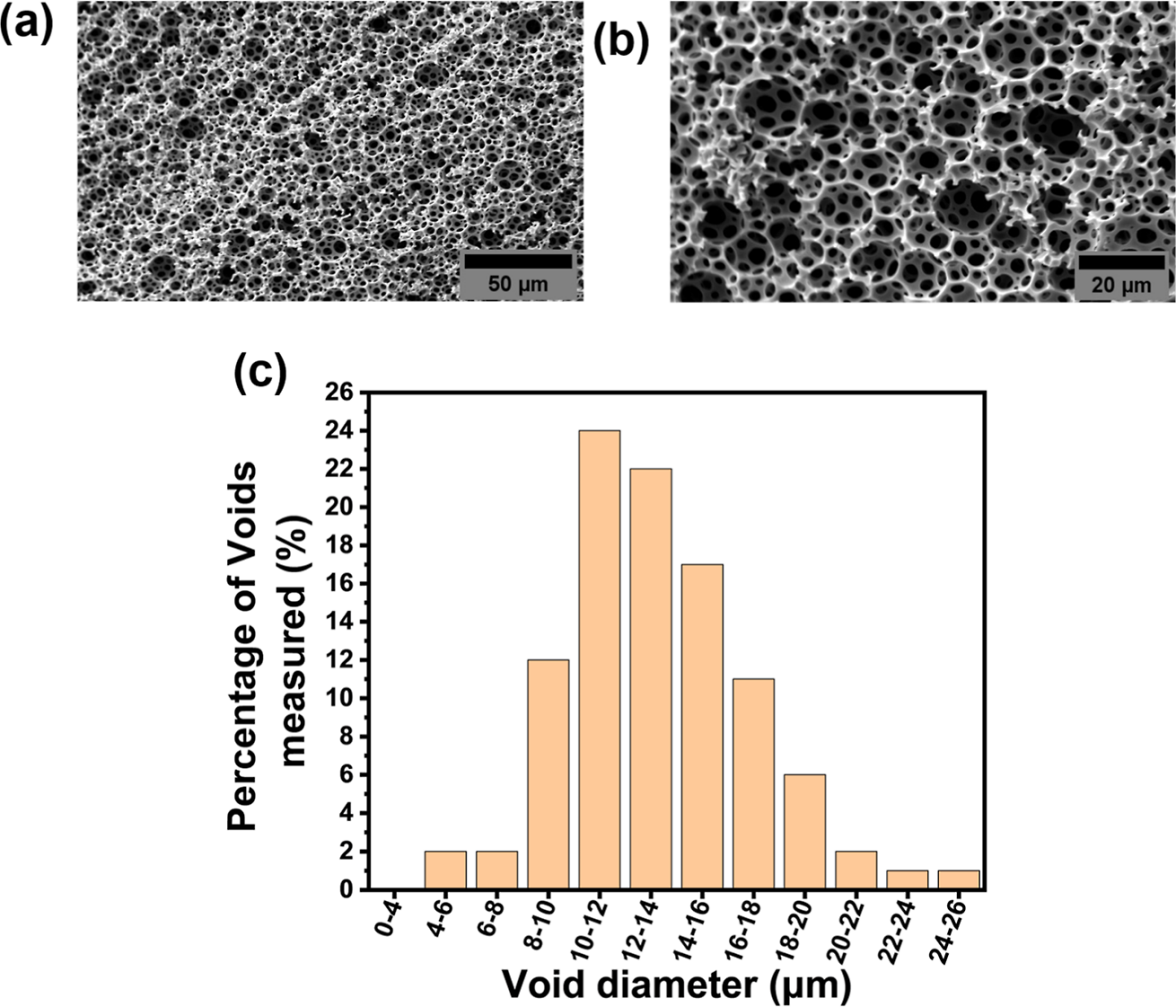
SEM image of GMA-PolyHIPE material at (A) 500x and (B) 1000× magnification. (C) Void diameter distribution for GMA polyHIPEs. 100 voids were measured and a correction factor of 2/(3^0.5^) was applied to account for the underestimation of void size.

**Figure 2:**
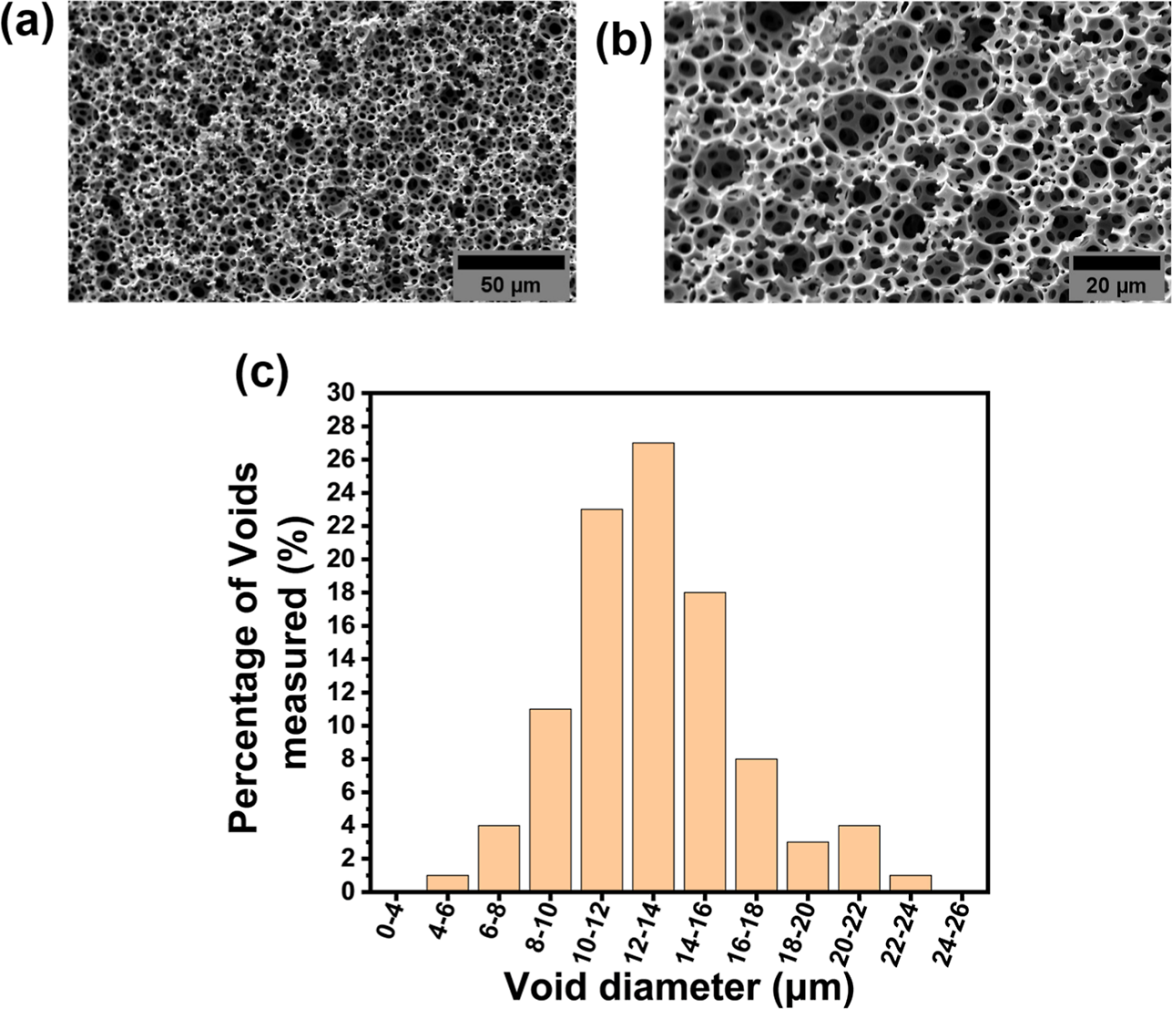
SEM image of N_3_-polyHIPE material at (A) 500× and (B) 1000× magnification. (C) Void diameter distribution for N_3_-polyHIPEs. 100 voids were measured and a correction factor of 2/(3^0.5^) was applied to account for the underestimation of void size.

FT-IR analysis (Figure 3) of GMA-polyHIPE showed characteristic C–O–C stretching peaks at ca. 910 and 845 cm^–1^, confirming the presence of surface epoxide groups. Following reaction with sodium azide, the FT-IR spectrum of N_3_-polyHIPE confirmed the successful introduction of azide functionalities, evidenced by the appearance of a peak at ca. 2100 cm^–1^ and a clear decrease in the intensity of the C–O–C peaks. The appearance of a broad O–H vibrational stretch at ca. 3400 cm^–1^ was also observed (Figure 3). The azidification reaction takes place via a nucleophilic attack of the azide to the epoxide ring, forming the regio-isomer shown in Scheme 1.^21^ The successful azidification provides a versatile chemical handle for the subsequent attachment of biomolecules, such as hyaluronic acid, to tailor the scaffolds bioactivity for stem cell culture.

**Figure 3:**
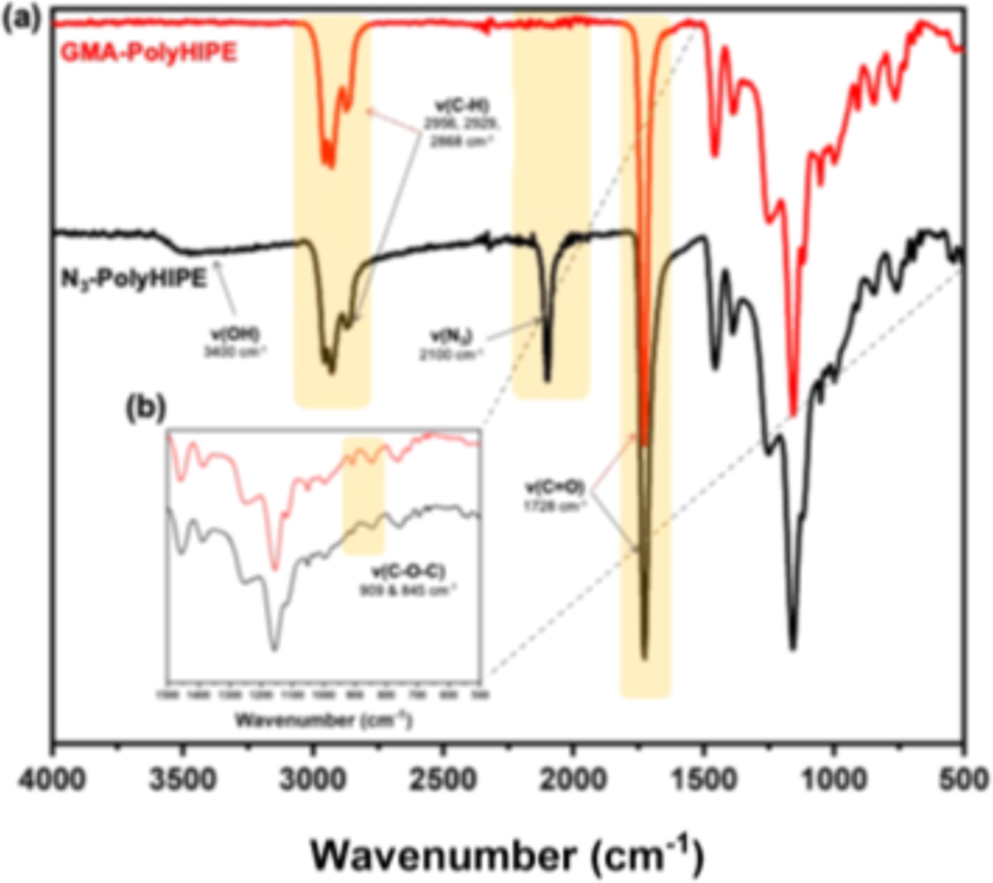
(A) FT-IR spectra of GMA- and N_3_- polyHIPE materials, respectively. (B) Fingerprint region highlighting the reduction of C–O–C epoxide peaks.

**Scheme 1:**
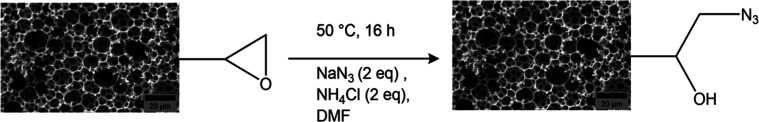
Azidification Reaction Conditions

Mechanical compression testing revealed similar Young’s modulus values for GMA-polyHIPE (20.0 ± 0.2 kPa) and N3-polyHIPE (19.7 ± 0.3 kPa) materials (Table 1), suggesting the azidification process did not significantly alter the mechanical strength. These values are comparable to other polyHIPE scaffolds used in 3D cell culture.^10,11^ This range of Young’s modulus is relevant as stem cells are sensitive to the mechanical cues of their environment, and such values may be suitable for certain stem cell types. While standard deviations are provided, a formal statistical analysis was not performed on this data due to the small sample size (*n* = 3). Therefore, the observation that the Young’s modulus remained similar is based on the trend observed.

**Table 1:** Young’s Modulus Values Recorded for Each polyHIPE Scaffold from Mechanical Compressions

	Young’s modulus (kPa)			
sample	sample 1	sample 2	sample 3	average ± SD
GMA-PolyHIPE	20	19.8	20.2	20.0 ± 0.2
N_3_-polyHIPE	19.7	19.5	20	19.7 ± 0.3
HA-polyHIPE (LSC)	21.4	18.4	14.7	18.0 ± 3.4
HA-polyHIPE (HSC)	19.1	13.1	21.1	17.8 ± 4.2

XPS elemental analysis and N 1s spectra also supported the successful azidification reaction. The high-resolution N 1s spectra showed no peak in the GMA-polyHIPE and a new peak at 400.4 eV in the N_3_-polyHIPE spectrum. The elemental surface composition indicated the appearance of nitrogen from 0 to 0.87% of the total elemental composition from unfunctionalized to functionalized scaffold. Given the well-established high efficiency of the epoxide-to-azide conversion and the subsequent click reaction, we infer a high degree of HA functionalization, with the surface coverage expected to be proportional to the initial epoxide functionality. This XPS data further confirms the presence of azide groups on the material surface, which is crucial for the next step of hyaluronic acid functionalization aimed at enhancing stem cell interaction.

The synthetic strategy for obtaining hyaluronic acid (HA)-functionalized polyHIPE (HA-polyHIPE) materials was based on a coupling reaction between N_3_-polyHIPE material and alkyne end-capped HA (HA-Alk) using the copper-catalyzed Huisgen cycloaddition “click” reaction. For that purpose, the alkyne functionality was introduced to the reducing end of HA by a two-step synthesis; HA activation by ion exchange followed by reductive amination of the activated HA with propargylamine (Scheme 2). Briefly, HA sodium salt was activated by a simple cation exchange of Na^+^ with tetrabutylammonium cation (TBA^+^). This was necessary to improve the solubility of HA in organic solvent (dimethyl sulfoxide, DMSO) for the reductive amination step. The reductive amination proceeded via an imine intermediate formation from the nucleophilic attack of propargyl amine to the carbonyl of the aldehyde functionality present at the chain end in the open-chain form. The imine intermediate is then reduced. Sodium cyanoborohydride is usually used as the reducing agent of choice, due to high selectivity and product yields.^69^ However, in this procedure, sodium triacetoxyborohydride was chosen to be used as a less toxic, easier to handle reducing agent alternative. The procedure was designed to ensure that the alkyne functionality is singularly coupled to the reductive end of HA, retaining chain integrity. Multipoint attachment along the HA backbone is not desirable, as this could influence HA chain conformations and alter interactions with CD44 cell receptors.^70,71^

**Scheme 2:**
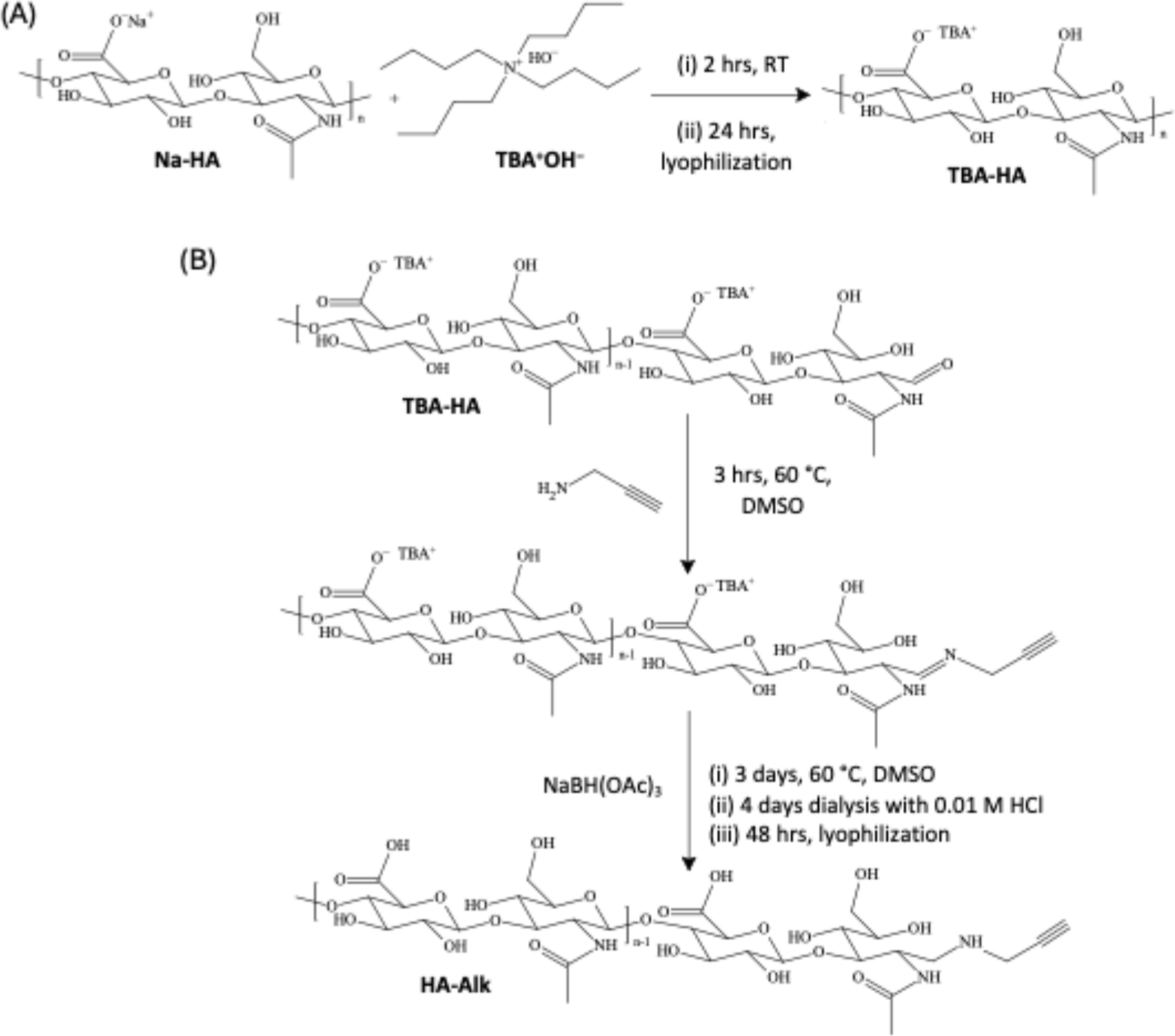
(A) Ion Exchange with TBA^+^ (B) Reductive Amination Reaction Pathway through Imine Intermediate

HA-Alk was fully characterized by NMR, FT-IR, Raman and XPS spectroscopy. Due to the relatively high molecular weight of the HA used, it was difficult to directly obtain prove the successful introduction of the propargyl functionality to HA from NMR and IR spectroscopies (Figures S2 and S3, Supporting Information). While the Raman spectrum of the HA-Alk showed a new peak at ca. 2260 cm^–1^, attributed to the C≡C vibrational mode (Figure S4, Supporting Information), it was quite small and therefore further evidence was required to prove the successful functionalization. The successful characterization of HA-Alk is critical to ensure the subsequent functionalization of the polyHIPE scaffold with a molecule known to interact with stem cells.

High-resolution C 1s and N 1s XPS spectra did provide strong evidence for the successful reductive amination reaction and introduction of an alkyne functionality to HA chain end. To identify the new C≡C bond, the C 1s spectra of HA and HA-Alk were deconvoluted using mixed Gaussian–Lorentzian (Voigt) line shapes (Figure 4A,B). The C 1s spectrum of HA showed six regions: C–C and C–H (285.0 eV), C–O (287.0 eV), C–N (285.7 eV), O=C–N (288.3 eV), O=C–O (288.5 eV) and O–CO–O (289.8 eV) and was used as a reference for deconvoluting HA-Alk. By overlaying and peak-fitting the two spectra (Figure 4C,D), a clear shoulder peak appeared in the HA-Alk spectrum that required an extra Gaussian proposed to correspond to the C≡C bond (284.1 eV). Analogous to NMR, different chemical environments can shift the core binding energies. sp^2^ and sp hybridized carbon atoms have been reported to shift to slightly smaller binding energies than sp^3^ hybridized carbon atoms.^72^ The percentage of this C≡C region (1.93%) was much higher than what would be expected as a relation to the number of other carbon bonds. This likely resulted from the attenuation of other regions associated with the HA polymer backbone, which without a counterion can undergo secondary structure rearrangements. The triple bond could be orientated in such a way on the HA-Alk surface that it appears more significant than the chemical composition would suggest. This detailed XPS analysis confirms the successful modification of HA with alkyne groups, enabling its subsequent covalent attachment to the azide-functionalized polyHIPE scaffold for enhanced stem cell interaction.

**Figure 4:**
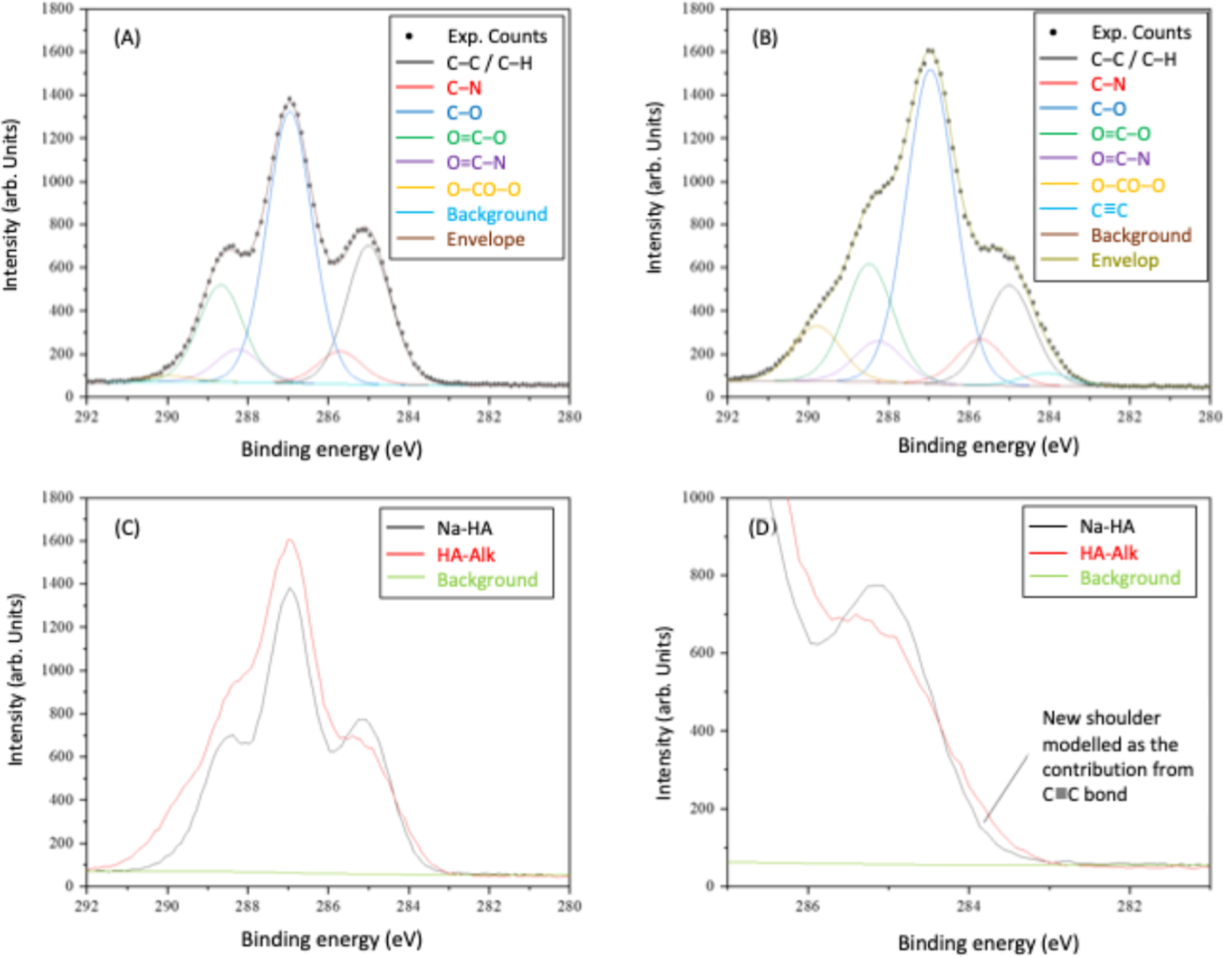
XPS high-resolution peak-fitted C 1s spectra for (A) Na-HA and (B) HA-Alk. (C) Overlaid C 1s spectra for Na-HA and HA-Alk with (D) showing the new shoulder.

Differences were also observed in the N 1s spectra of HA and HA-Alk as shown in Figure 5. The HA spectrum showed only one component (400.4 eV) which was assigned to the amide bonding environment from the *N*-acetylglucosamine unit. Upon reductive amination and addition of the alkyne functionality, deconvolution revealed a second smaller peak was required to fit the higher energy shoulder at 401.9 eV (Figure 5C). This new peak was found to be at a higher percentage of the overall region than expected (17.5%). As previously explained, this is likely to be due to the same attenuation effect observed in the C 1s spectra. The changes in the N 1s spectra further support the successful introduction of the alkyne group to HA, a modification necessary for its immobilization onto the polyHIPE scaffold to potentially improve stem cell adhesion and growth.

**Figure 5:**
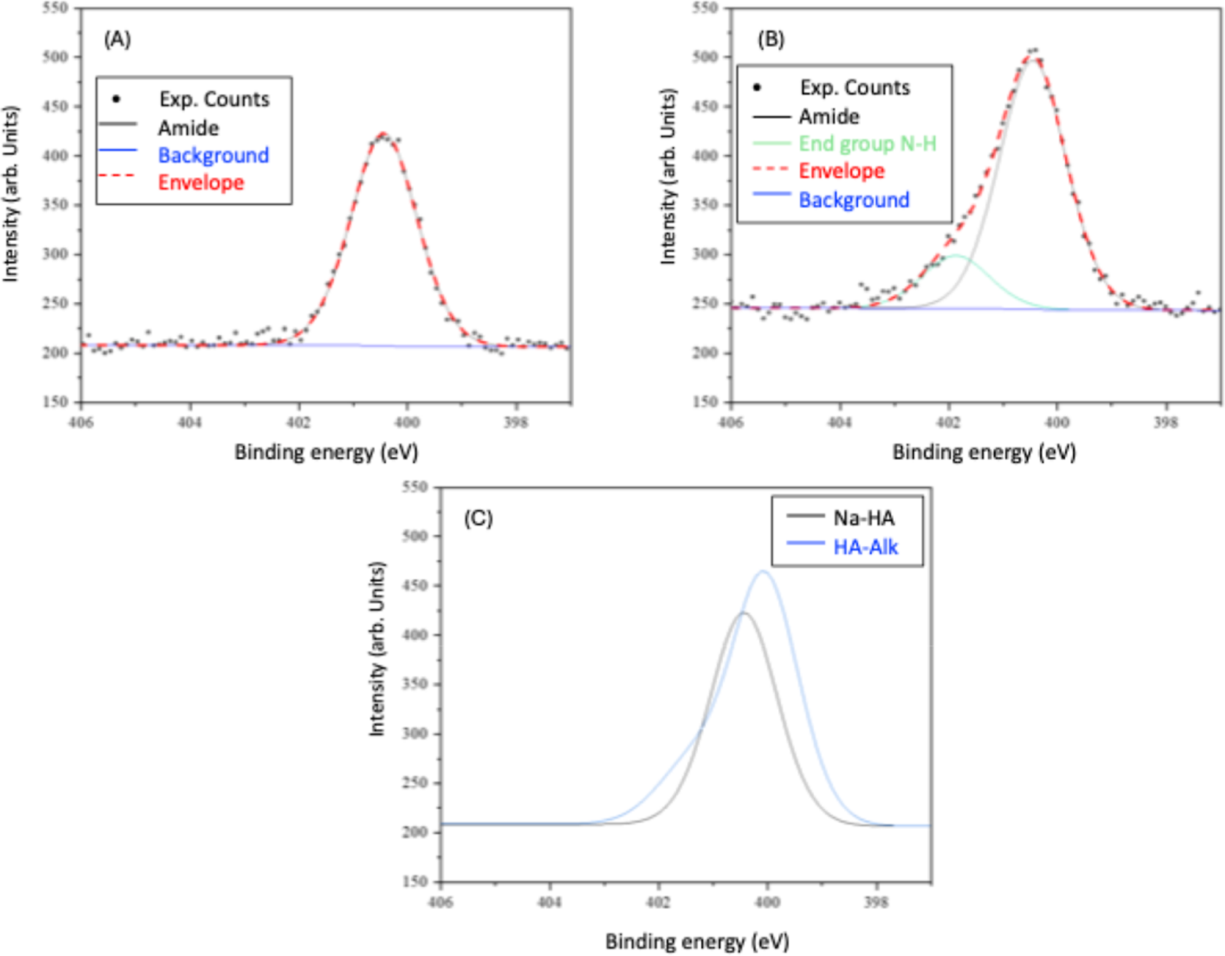
XPS high-resolution peak-fitted N 1s spectra for (A) Na-HA and (B) HA-Alk. (C) Overlaid fitted N 1s spectra for Na-HA and HA-Alk.

Additionally, the elemental analysis identified a decrease in the carbon-to-nitrogen ratio from 18.5 in HA to 16.9 in HA-Alk, consistent with the addition of an amine group (Table S1, Supporting Information). Similar to the individual spectral peaks, this was also abnormally high but can be explained again by the attenuation effect. The elemental analysis provides further quantitative evidence for the successful chemical modification of HA, which is a prerequisite for creating a bioactive scaffold for stem cell culture.

Prior to conducting the click reactions between HA-Alk and N_3_-polyHIPE material, reaction conditions were optimized for the small molecules, propargyl alcohol (PrOH) and propargyl amine (PrNH_2_), Scheme 3. Previous Copper-catalyzed Alkyne Azide cycloaddition (CuAAC) reactions with polyHIPE material have used copper­(I) salts, as these systems often require lower catalyst loading and reaction times.^21^ However, in this work, it was chosen to use a Cu­(II) salt system as these systems are more resistant to oxidation from air or aqueous solvents and readily produce the reactive Cu­(I) species in situ upon addition of reducing agent, e.g. Na-Ascorbate.^73^ As a result, no anhydrous, deoxygenated solvents are required and fewer side products are potentially formed, at the minor cost of increased reaction time and catalyst loading. A range of solvent systems were tested, including dimethyl sulfoxide (DMSO), iso-propyl alcohol (IPA) and *tert*-butanol. A 1:1 mixture of *tert*-butanol to water was found to be the optimum solvent ratio for balancing maximum penetration/swelling of polyHIPE material with dissolving the hydrophilic HA-Alk or small molecule (PrOH/PrNH_2_), copper­(II) salt and reducing agent. Optimizing the click reaction conditions on small molecules is a crucial step to ensure efficient and successful immobilization of hyaluronic acid onto the polyHIPE scaffold for subsequent stem cell culture studies.

**Scheme 3:**
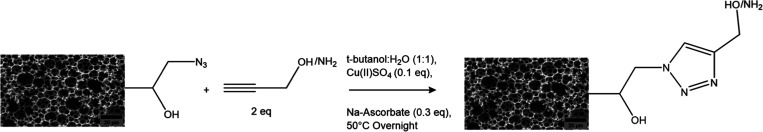
Reaction Procedure for Click Reaction with Small Molecules

FT-IR spectra of both polyHIPE materials clicked with PrOH and PrNH_2_ showed almost complete disappearance of the azide peak at 2100 cm^–1^, indicating approximately quantitative conversion to the clicked products (Figures S5 and S6, Supporting Information). Also, appearance of new peaks at 3274 and 761 cm^–1^ corresponding to the N–H stretching and primary amine wagging of the amine functionality, respectively, in the FT-IR spectrum of the polyHIPE material clicked with PrNH_2_ (Figure S5, Supporting Information) as well as the increased vibrational intensity of the −O–H peak at 3347 cm^–1^ as a result of the extra hydroxyl functionalities in the FT-IR spectrum of the polyHIPE material clicked with PrOH (Figure S6, Supporting Information) support the successful click reactions. This confirms the efficacy of the click chemistry approach for surface modification, which was subsequently applied to immobilize hyaluronic acid, a key component for influencing stem cell behavior.

Further visual confirmation of the successful click reaction was achieved by reacting an N_3_-polyHIPE scaffold with an alkyne functionalized dye, carboxyrhodamine-110, Figure S7, Supporting Information, under the same reaction conditions used earlier. Prior to taking its photograph (Figure S8, Supporting Information), the produced polyHIPE scaffold was extensively washed with methanol and dried to ensure complete removal of any residual unreacted dye. This visual confirmation reinforces the reliability of the click chemistry method used for the crucial step of attaching hyaluronic acid to promote stem cell interaction.

Lastly, HA was successfully clicked onto the polyHIPE surface using similar reaction conditions used for small molecules. Different HA-Alk to polyHIPE w/w ratios (1:1, 10 mg:10 mg low surface concentration, LSC); 1:2, 10 mg:20 mg; and 1:3, 10 mg:30 mg (high surface concentration, HSC) were used. The FT-IR spectra of the starting material N_3_-polyHIPE as well as HA clicked products are shown in Figure S9, Supporting Information. Four key regions in the spectra indicate the successful attachment of HA: (1) increase in the intensity of the broad OH peak at 3347 cm^–1^; (2) decrease in the intensity of the azide peak at 2100 cm^–1^; (3) appearance of two new peaks corresponding to the amide stretches (1636 and 1558 cm^–1^); and (4) appearance of new peaks corresponding to the HA polymer backbone (1074 and 1049 cm^–1^). The increase in intensity of the broad peak at 3347 cm^–1^ is attributed to the OH groups along the HA polymer chain. The consumption of azide functionality suggests transformation to the potential click product. The azide peak reduction was not expected to be significant due to the very low equivalence used. The two new peaks observed at 1636 and 1558 cm^–1^ are attributed to the amide stretches for C–O and N–H, respectively, in the HA-Alk. Finally, significant increases proportional to higher weight ratios for stretches at 1074 and 1049 cm^–1^ relate to a variety of vibrational motions characteristic of the HA polymer backbone. Taken together, these peak changes have demonstrated the successful click reaction and chemical attachment of HA to the polyHIPE surface. This spectroscopic evidence confirms the successful functionalization of the polyHIPE scaffold with hyaluronic acid, a crucial step toward creating a bioactive material that can potentially enhance stem cell adhesion, proliferation, and differentiation.

SEM and mechanical testing were also conducted on the clicked polyHIPE scaffolds to determine if any changes were observed. SEM micrographs confirmed that the open-cell porous morphology remained unchanged with an average pore diameter of 15.9 ± 4.5 μm, Figure 6. The maintenance of this porous structure after HA functionalization is important for ensuring continued stem cell infiltration and nutrient transport. Mechanical testing for each weight ratio showed a slight reduction in Young’s modulus values for HA-polyHIPE compared to N_3_-polyHIPE and GMA-polyHIPE materials, Table 1. It is believed that this change in the mechanical strength of the materials is not significant and not expected to affect the application of the material in cell culture. The slightly altered mechanical properties after HA attachment may influence stem cell behavior at the material interface, and further studies could explore this relationship. However, the overall mechanical stability of the scaffold is maintained.

**Figure 6:**
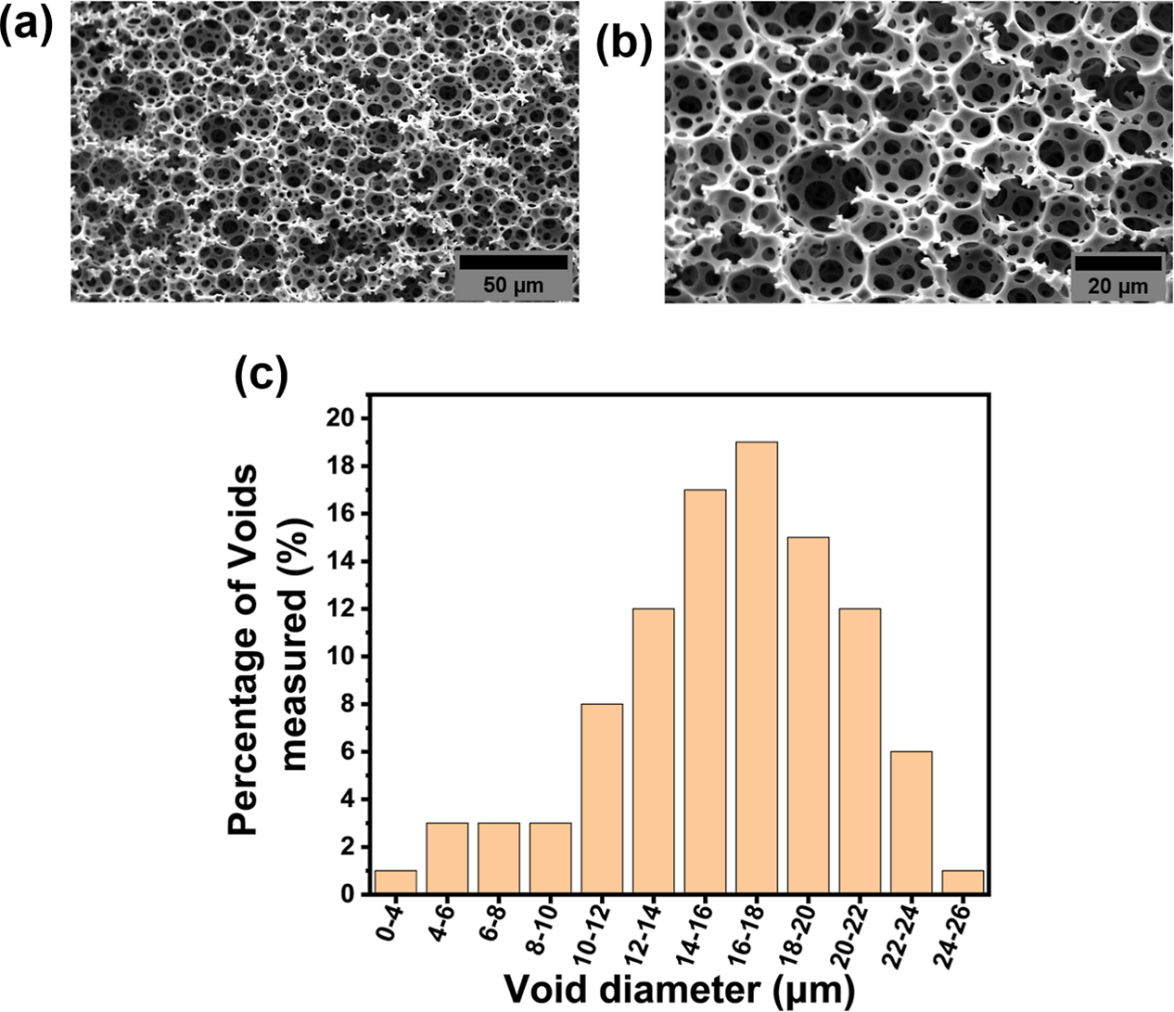
SEM micrographs of HA-polyHIPE material at HSC, taken at (A) 500× and (B) 1000× magnification. (C) Void diameter distribution. 100 voids were selected and a correction factor of 2/(3^0.5^) was applied to account for the underestimation of void size.

High-resolution XPS was applied to the resulting HA-polyHIPE scaffolds and compared with the N_3_-polyHIPE and GMA-polyHIPE starting materials. As shown in Figure 7, N 1s spectra show an increase in the nitrogen peak for the HA-polyHIPE samples. This is due to the fact that HA contains extra nitrogen atoms from the amide groups along the polymer chain. Elemental analysis also showed an increase in the percentage nitrogen for the HA-polyHIPE LSC (1.13%) and HSC (1.36%) compared to the N_3_-polyHIPE scaffolds (0.87%), Table 2. The increased nitrogen content observed in XPS confirms the presence of the nitrogen-containing amide groups of hyaluronic acid on the scaffold surface, further supporting the successful functionalization aimed at enhancing stem cell interaction.

**Figure 7:**
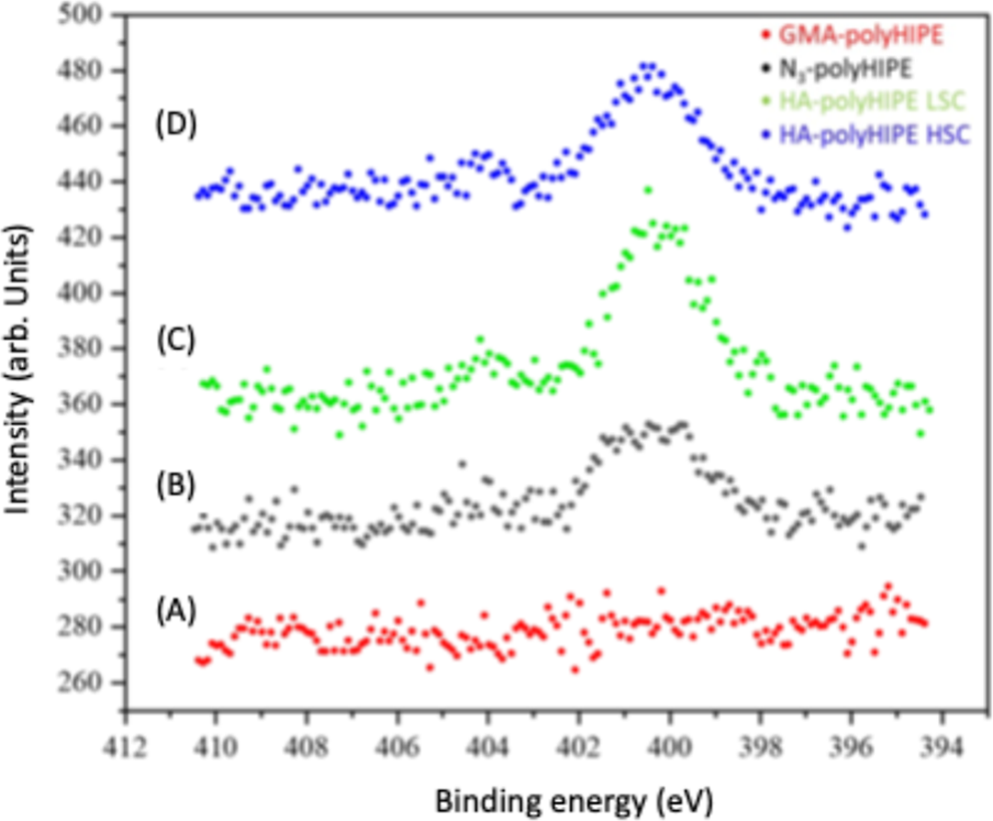
High-resolution XPS spectra for (A) GMA-polyHIPE (B) N_3_-polyHIPE (C) HA-polyHIPE LSC (D) HA-polyHIPE HSC.

**Table 2:** XPS Elemental Composition Analysis for GMA-PolyHIPE, N_3_–PolytHIPE and HA-polyHIPE LSC & HSC

sample	C 1s	O 1s	N 1s	P 2p	Si 2p
GMA-polyHIPE	80.56	18.55		0.47	0.43
N_3_-polyHIPE	78.44	18.37	0.87	0.11	2.21
HA-polyHIPE LSC	78.26	17.62	1.36	0.1	2.66
HA-polyHIPE HSC	73.79	20.92	1.13	0.13	4.04

### 2.2 Scaffold-Based Cell Culture

The potential of hyaluronic acid-functionalized polyHIPE scaffolds to support the growth and expansion of CD34^+^ cells was investigated in preliminary culture tests. The scaffolds were seeded statically, as previously described (Severn et al.,[Bibr ref12] 2019), using 0.5 × 10^6^ CD34^+^ cells per scaffold isolated from peripheral blood mononuclear cells (PBMNCs). The cells were cultured in StemSpan SFM medium supplemented with a growth factor cocktail for 20 days, with complete medium changes every 2 days. Cell proliferation within the scaffold was assessed by measuring the number of cells that migrated out of the scaffold into the surrounding medium, termed cell egress, which was quantified using flow cytometry.

The results showed that the peak of cell egress occurred on day 12 of the culture, Figure 8A. By the end of the 20 day culture period, the total number of egressing cells was comparable between the LSC HA-polyHIPE (2.35 × 10^6^ cells) and the HSC HA-polyHIPE (2.47 × 10^6^ cells) scaffolds. While the HA-polyHIPE scaffolds showed a trend of outperforming control cultures (GMA- and N3-polyHIPE scaffolds) in terms of cell egress (averaging 2.41 × 10^6^ cells compared to 2 × 10^6^ cells, Figure 8B), formal statistical significance was not determined for these results. The error bars in Figure 8B illustrate the variability within the data sets. A more rigorous statistical analysis will be performed in future studies with increased replicates to confirm the observed trends. This enhanced proliferation is likely due to the combined effects of the polyHIPE scaffold’s structure and the HA functionalization. The highly porous structure of the polyHIPE provides a large surface area for cell attachment and facilitates efficient nutrient and waste exchange, creating a favorable environment for cell growth. Furthermore, the HA modification introduces specific biological cues; HA is known to interact with cell surface receptors such as CD44, which are present on CD34^+^ cells, potentially promoting cell adhesion, survival, and proliferation signaling. Additionally, HA’s hydrophilic nature can improve the scaffold’s wettability, aiding in cell seeding and distribution within the 3D structure. This suggests that the HA-polyHIPE scaffolds can support cell growth and more work is needed to understand how the cells grow within the scaffolds. Cell egress populations were analyzed using a flow cytometry panel on days 8, 12, 16, and 20 of the culture, Figure 8C. The panel included CD34, CD36, CD61, CD117 and GPA surface markers. The mean fluorescence intensity of flow cytometry data can be found in Figure S10, Supporting Information. CD34^+^ stem cells, a marker of hematopoietic stem and progenitor cells (HSPCs), showed a persistent population throughout the culture period, suggesting that the HA-polyHIPE scaffolds support the preservation of the stem/progenitor cell population. Similar to the results previously observed by Severn & Eissa et al.,^12^ CD36 expression, a marker expressed on various cell types including platelets and some erythroid precursors, remained stable throughout the culture period, highlighting the sustained presence of megakaryocyte and erythroid progenitors (MEPs), indicating the early stages of myeloid and erythroid lineage commitment. In contrast, CD61 expression, a marker found on platelets and megakaryocytes, in scaffold egress cells remained moderate but consistent from day 8 to day 20, further supporting the presence of megakaryocyte lineage cells. CD117 expression, a receptor tyrosine kinase expressed on hematopoietic stem cells and other cell types, decreased over the 20 day period culture, indicating a reduction in the more immature myeloid cell population over time. On the other hand, glycophorin A (GPA) expression, a marker specific to erythroid cells, steadily increased, suggesting a rise in the proportion of cells committed to the erythroid lineage. Overall, these findings indicated that HA-functionalized polyHIPE scaffolds can support the proliferation of CD34^+^ cells while also allowing for differentiation along multiple hematopoietic lineages when compared to the controls.

**Figure 8:**
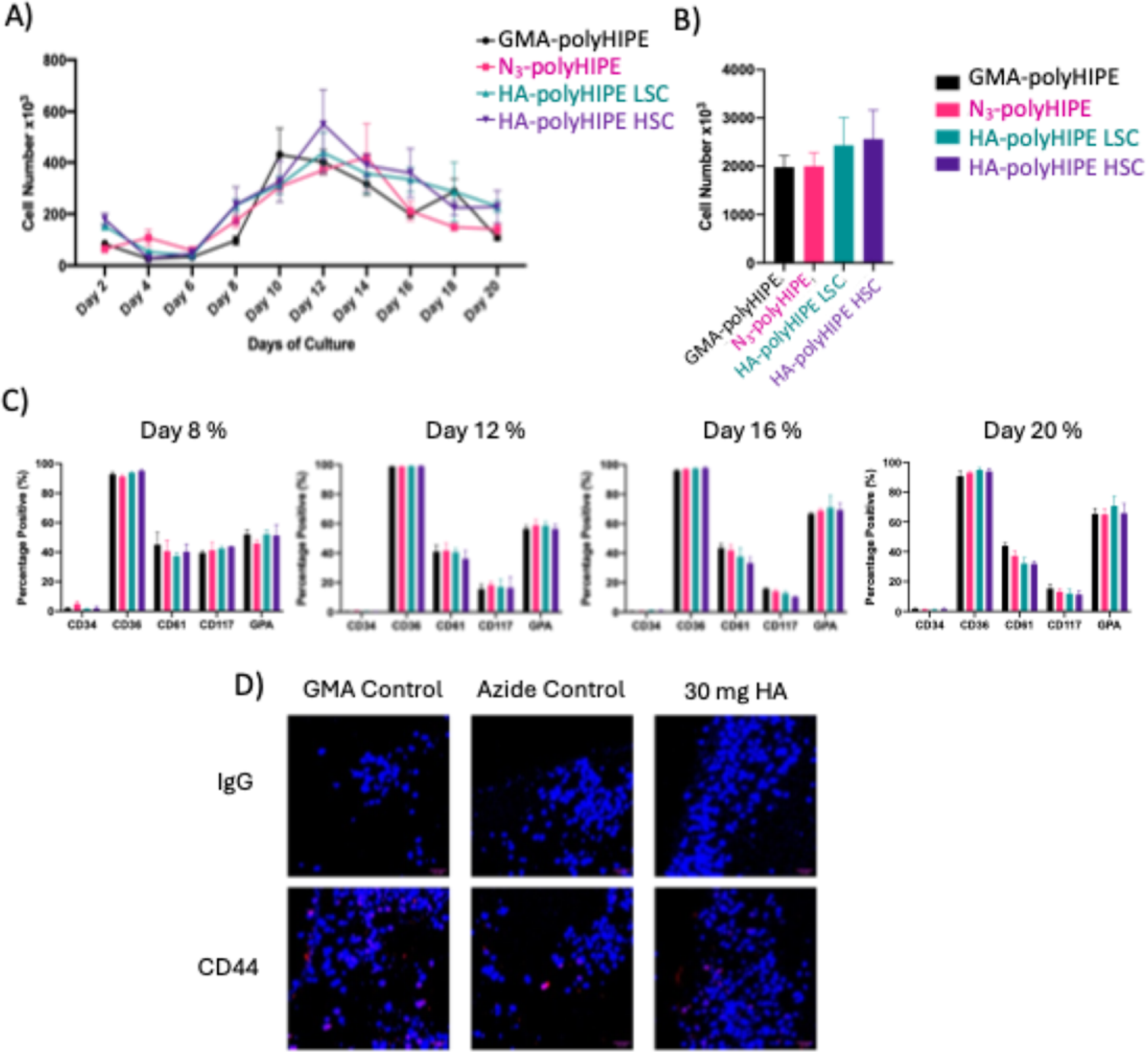
3D cell culture experiments showing that HA-polyHIPE scaffolds can maintain and promote growth of CD34^+^ cells. MACSQuant flow cytometer with automated addition of propidium iodide was used to count and discriminate live from dead cells. Cellular egress from GMA-, Azide-, HA- and HA-functionalized polyHIPE (LSC and HSC) scaffolds is shown as number or percentage of the total cell live population. (A) Cellular egress counts every 2 days over a 20 day period. (B) Cumulative number of cellular egress after 20 day culture. (C) Cell phenotype of cellular egress conducted by flow cytometry analysis on days 8, 12, 16, and 20. (D) Leica SP5 confocal microscopy images at 63× magnification were taken from 10 μm paraffin sections of GMA control, azide control, and HSC HA-functionalized polyHIPE scaffold samples. DAPI (blue) was used for nuclear staining, while IgG or CD44 is shown in red.

CD44 labeling on paraffin embedded scaffolds confirm occupation by progenitors within the scaffold, suggesting that HA-functionalized polyHIPEs facilitate the culture of cells within the scaffold material, Figure 8D.

## 3 Conclusions

HA was successfully modified and attached to the surface of polyHIPE materials, via CuAAC click chemistry. This was achieved in four steps; synthesis of photopolymerized GMA-based polyHIPEs, azidification of the surface epoxide groups to form an N_3_-polyHIPE, HA functionalization via reductive amination to form HA-Alk, finally followed by a CuAAC reaction between N_3_-polyHIPE scaffolds and HA-Alk. The photopolymerized GMA-based polyHIPE materials exhibited robust mechanical properties, with a Young’s modulus of 20.0 ± 0.2 kPa which is important for providing structural support to cells. SEM analysis revealed an interconnected, porous structure characteristic of polyHIPEs, with a calculated pore diameter of 13.3 ± 3.7 μm, a feature crucial for facilitating cell infiltration and nutrient transport. FT-IR spectra confirmed successful azidification through the appearance of an azide peak at 2100 cm^–1^ and the reduction of C–O epoxide stretches around 900 cm^–1^. Importantly, azidification did not compromise the material’s mechanical or porous properties, ensuring the scaffold’s structural integrity is maintained during functionalization. Future studies could explore the effects of different GMA concentrations on material morphology and porosity.

HA was successfully functionalized with an alkyne group using reductive amination. The large size of HA made it difficult to obtain direct spectroscopic evidence using conventional ^1^H NMR and FT-IR methods. However, ^1^H NMR spectroscopy confirmed quantitative ion exchange from Na^+^ to TBA^+^. Raman and high-resolution XPS analysis provided evidence of successful functionalization. Raman spectra showed a tentative new peak at 2260 cm^–1^ attributed to the alkyne stretch. XPS C 1s and N 1s spectra exhibited shoulders indicative of new functional groups (amine and alkyne).

Small model molecules, PrOH and PrNH_2_, served as precursors for the CuAAC click reaction with HA-Alk. FT-IR experiments confirmed successful azide reduction, indicating a complete reaction. Future optimization efforts could focus on exploring a wider range of solvent systems to enhance reagent and biomolecule diffusion within the material’s core. HA was subsequently clicked onto the polyHIPE surface at varying weight concentrations. FT-IR and XPS data confirmed HA attachment, with new characteristic peaks appearing in FT-IR spectra and an increased N 1s peak intensity in XPS spectra, demonstrating the successful modification of the polyHIPE surface with HA.

Scaffold-based cell culture experiments demonstrated that HA-functionalized polyHIPE scaffolds can support the maintenance of stemness (CD34^+^) and facilitate the proliferation of hematopoietic cells (CD36, CD61, CD117, GPA positive cells) over a 20 day culture period. HA-functionalization did not adversely affect cell viability or disrupt the polyHIPE honeycomb structure, maintaining the properties of polyHIPEs as previously characterized for hematopoietic stem cells (HSCs) and mesenchymal stem cells (MSCs), highlighting the biocompatibility of the modified scaffolds and their ability to support stem cell culture. The results unequivocally indicate that HA-functionalized polyHIPE scaffolds possess excellent biocompatibility and support stem cell culture, suggesting a substantial level of HA incorporation onto the polyHIPE surface. Future studies should investigate the impact of HA density on cell proliferation and occupancy rates to optimize the scaffold for enhanced stem cell expansion. The conclusions drawn from our experiments are based on observed trends and descriptive statistics (mean, standard deviation, error bars).

While this study demonstrates the successful initial functionalization of the polyHIPE scaffolds with HA, the durability and stability of this surface modification over extended periods in culture conditions were not directly evaluated. Therefore, future studies should also investigate the longevity of the HA functionalization to fully assess their potential for long-term applications.

To further advance cell culture applications, these HA-functionalized polyHIPE scaffolds will next undergo rigorous in vitro biological assessments, including long-term cell viability assays and detailed differentiation studies, with statistical analysis on increased sample sizes and experimental replicates to confirm the significance of the observed trends in mechanical properties and cell behavior, building upon our previous work and the established methodologies for 3D hematopoietic stem cell culture.^12,74^ Specifically, future work should prioritize in-depth biological assessments, including long-term cell viability assays to evaluate extended culture periods, detailed differentiation studies to assess the impact of the HA-polyHIPE scaffolds on stem cell fate, and in vivo studies to explore the potential for tissue regeneration. Moreover, alkyne functionalization could be explored as a promising versatile precursor for polyHIPE click chemistry with other glycosaminoglycans such as chondroitin sulfate, dermatan sulfate, heparin, and heparan sulfate, opening up avenues for creating scaffolds with tailored bioactivity for various tissue engineering applications with the ultimate goal of developing clinically relevant scaffolds for regenerative medicine.

## 4 Experimental Details

### 4.1 Materials

The chemicals used were obtained from Sigma-Aldrich or Fischer Scientific at TG unless stated otherwise. 2-Ethylhexyl acrylate (EHA; ⩾ 98%), glycidyl methacrylate (GMA; 97%), hypermer B246 (triblock copolymer of poly­(12-hydroxystearic acid), isobornyl acrylate (IBOA), trimethylolpropane triacrylate (TMPTA; TG), diphenyl­(2,4,6-trimethylbenzoyl)­phosphine oxide/2-hydroxy-2-methylpropiophenone blend (photoinitiator), dichloromethane (DCM), *N*,*N*-dimethylformamide (DMF), Sodium Azide (NaN_3_; 99%), ammonium chloride (NH_4_Cl; TG), propargyl alcohol (PrOH).; 99%), propargyl amine (PrNH_2_; 99%), hyaluronic acid sodium salt (Na-HA, MW 8000–16,000, Carbosynth), tetrabutylammonium hydroxide in water (1.5 M, TBA), dimethyl sulfoxide (DMSO), sodium triacetoxyborohydride (NaBH­(OAc)_3_), hydrochloric acid (0.01 M, HCl), carboxyrhodamine-110 (>95%, Click Chemistry Tools), tert-butanol. Dowex 50WX8–400 resin was used for TBA activation.

### 4.2 Instrumentation and Characterization

Infra-Red spectra were collected using a Bruker Alpha FT-IR Spectrometer in a range of 400–4000 cm^–1^ and processed using OPUS software. Raman spectra were collected with a Horiba LabRam HR instrument for an acquisition time of 25 s. A 660 nm Laser at 50% power (nominally 20 mW) was used with a grating of 600 lines per mm and confocal lens with objective ×50 LWD and hole size of 150 μm. Results were normalized, baseline corrected, fitted with a fifth order polynomial and denoised with the algorithm contained in the software LabSpec 6.

Single compression tests were conducted with 2 to 4 cubes (**A12**), with dimensions approximately 0.5 × 0.5 × 0.5 cm, using a Shimadzu EZ-LX with a load capacity of 500 N and plate speed of 20 mm/min. TAPEZIUM X software was used for calibration and data extraction. SEM images were collected via a Philips/FEI XL30 ESEM operating at 25 kV. Average void diameters were calculated using ImageJ software. A total of 100 void diameters were randomly chosen and calculated from an SEM image at 1000× magnification. A statistical correction factor then was applied to account for an underestimation of the true value, due to the unlikely chance of exact bisection.^75^ ^120^

^1^H 1D NMR spectra were collected using a Bruker Advance III 400 Spectrometer operating at a frequency of 400.1 MHz and analyzed with ACD laboratories 12.0.

The XPS measurements were collected at the Warwick Photoemission Facility using a Kratos Axis Ultra DLD spectrometer. The samples investigated in this study were attached to electrically conductive carbon tape, mounted onto a sample bar with a layer of filter paper between the samples and the sample bar to ensure electrical isolation, before being loaded in. The experiment was performed in the main analysis chamber, with each sample being illuminated using a monochromated Al Kα X-ray source (*h*ν = 1486.7 eV). The measurements were conducted at RT and a takeoff angle of 90° with respect to the surface parallel. The core-level spectra were recorded using a pass energy of 20 eV (resolution approximately 0.4 eV), from an analysis area of 300 μm × 700 μm. The work function and binding energy scale of the spectrometer were calibrated using the Fermi edge and 3d_5/2_ peak recorded from a polycrystalline Ag sample prior to running the experiments. To prevent surface charging, the surface was flooded with a beam of low energy electrons throughout the experiment, necessitating the recalibration of the binding energy scale. To achieve this, the main C–C/C–H component of the C 1s spectrum was referenced to 285.0 eV. The data were analyzed in the CasaXPS package, using Shirley backgrounds and mixed Gaussian–Lorentzian (Voigt) lineshapes. For compositional analysis, the analyzer transmission function was determined using clean metallic foils to obtain the detection efficiency across the full binding energy range.

### 4.3 Synthesis of GMA-Based polyHIPE Material

The formulation to prepare 25% v/v GMA-based polyHIPEs (GMA-PH) was based on work from Cameron et al. with a nominal porosity of 89% based on the aqueous phase content.^24,25^ An oil phase containing surfactant (0.2 g, 3% w/w of oil phase) was stirred in the dark at 350 rpm with an overhead stirrer in a 250 mL two-necked RBF. The oil phase contained EHA (2.72 g, 14.8 mmol, 40 mol %), GMA (1.87 g, 13.2 mmol, 36 mol %), TMPTA (1.41 g, 4.8 mmol, 13 mol %) and IBOA (0.87 g, 4.2 mmol, 11 mol %). Photoinitiator (0.87 mL, 10% v/v) was slowly added until the solution homogenized. An aqueous phase of 63 mL deionized water was added dropwise at a rate of one drop per second and further stirred 10 min. The HIPE was then transferred between two glass plates in a PTFE cube mold (0.5 × 0.5 × 0.5 cm) and passed under a UV irradiator four to six times on each side. The cured polyHIPE was washed by immersion in acetone overnight and dried under reduced pressure (RP) at ambient temperature for 2 days. Compression testing, FTIR spectrum and SEM images were obtained.

### 4.4 Azidification of GMA-Based polyHIPE Material

The ring-opening of surface epoxide groups from GMA-PH with sodium azide (NaN_3_) was based on a procedure from Heise et al.^21^ Briefly, for 200 mg of GMA-PH cubes, 20 mL of DMF was added to a 50 mL RBF and a mini stirrer bar. Sodium azide, NaN_3_ (580 mg, 9.5 mmol) and ammonium chloride NH_4_Cl (479 mg, 9.5 mmol) were added and stirred at 50 °C overnight. The azide-polyHIPEs (N_3_–PH) were washed thoroughly with water, then ethanol and left to dry under RP at ambient temperature for 1 day.

### 4.5 Reductive Amination of Hyaluronic Acid (HA)

The reductive amination of HA with propargyl amine was based on procedures from Li et al.^71^ and Hahn et al.^70^ Initially, Na-HA was activated with TBA via Dowex ion-exchange resin. Per 2 g batch of Na-HA, 20 g of resin was first washed three times with water (250 mL) before adding TBA (49 mL) and stirring for 30 min. The activated resin was collected by vacuum filtration and added to Na-HA dissolved in 100 mL of water. The reaction mixture was stirred at room temperature for 3 h, when the supernatant (TBA-HA) was filtered by 0.45 μm and lyophilized for 48 h to produce a white powder.

TBA-HA (2 g) was fully dissolved in DMSO (25 mL) and PrNH_2_ (96 μL, 5 equiv) was added and then stirred at 60 °C for 2 h. NaBH­(OAc)_3_ in 50 mL DMSO was added dropwise over a period of 20 min. Subsequently stirred at 60 °C for 3 days. Dialyzed with HCl (0.01 M) for 4 days then lyophilized for 48 h to produce a white powder. An approximate yield of 38% was obtained, due primarily to dialysis losses.

### 4.6 General N3-PH Click Reaction Procedure

The generalized procedure for successful click reactions with PrOH, PrNH_2_, dye and Alk-HA is briefly outlined. N_3_–PH (1 eq., 10 mg/cube, 1.88 × 10^–3^ mmol/cube) was soaked in excess tert-butanol. Alk-HA (10, 20, 30, 100 mg), PrOH/PrNH_2_ (10 equiv) or carboxyrhodamine-110 (1 mg) was dissolved in minimal water and added to a 20 mL vial. Cu­(II)­SO_4_ (0.1 equiv) in water was added to the vial. Tert-butanol was added dropwise with sonication for a final 1:1 ratio with water. Finally, the soaked cubes were transferred to the vial, passed over with nitrogen and sodium ascorbate (0.3 equiv) in water was added. The reaction was stirred at 60 °C overnight. The cubes were worked up with water, then ethanol washes and left to dry under RP at ambient temperature for 1 day.

### 4.7 Peripheral Blood Mononuclear Cell and CD34^+^ Cell Isolation

Peripheral blood mononuclear cells (PBMNC) were isolated from platelet apheresis blood waste (NHSBT, Bristol, UK) from healthy donors with informed consent. Ethics approval for all experimental protocols was granted by Bristol Research Ethics Committee (REC 12/SW/0199), and all methods were carried out in accordance with approved guidelines. PBMNC separation was performed using PBMNC Spin Media (PluriSelect) as described previously.^76,77^ CD34^+^ cells were isolated from PBMNCs using magnetic activated cell separation (MACS, Miltenyi Biotec) as previously described and according to manufacturer’s instructions.^77–79^

### 4.8 Scaffold Preparation and Three-Dimensional Erythroid Culture

Scaffolds were prepared and cultured as previously described.^12,74^ Briefly, scaffolds were prepared for culture by prewetting in phosphate buffered saline (PBS) for 20 min before the removal of PBS and then exposed to ultraviolet (UV) radiation for 15 min. The PBS was replaced with 70% ethanol for 2 h at room temperature with rotation. Finally, the scaffolds were washed twice with PBS to remove residual ethanol and stored in StemSpan SFM (Stem Cell Technologies) with 10% fetal calf serum (FCS, Gibco) and penicillin/streptomycin at 100 U/0.1 mg per mL of media respectively (Sigma), for at least 2 days to equilibrate at 37 °C with 5% CO_2_.

Scaffolds were dried of storage media using sterilized 3MM Whatmann paper and equilibrated to 37 °C until cell seeding. 0.5 × 10^6^ CD34^+^ cells were seeded statically in 20 μL of media, as described below. The scaffolds were then incubated at 37 °C with 5% CO_2_ for 2 h; with media added as required to ensure scaffolds did not dry out. 1.5 mL culture media consisting of StemSpan supplemented with penicillin/streptomycin at 100 U/0.1 mg per mL of media respectively (Sigma), cholesterol-rich lipids 40 μg/mL (Sigma), stem cell factor 100 ng/mL (SCF, Miltenyi Biotec), Interleukin-3 1 ng/mL (IL-3, R&D Systems), insulin like growth factor-1 40 ng/mL (IGF-1, R&D Systems), dexamethasone 1 μM/mL (Dex, Sigma) and erythropoietin 2 U/mL (Bristol Royal Infirmary) was gently added to each scaffold. Full medium changes were performed every 2 days. Scaffold egress was counted using the MACSQuant flow cytometer (Miltenyi Biotec). Cells were autolabeled with propidium iodide (Miltenyi Biotec) at a final concentration of 1 μg/mL. Dead cells were excluded, and a live cell count taken as a density per milliliter.

### 4.9 Flow Cytometry Panel

Flow cytometry was performed using 1 × 10^5^ cells labeled with extracellular conjugated antibodies for 30 min at 4 °C. Data were collected using a MacsQuant flow cytometer (Miltenyi Biotec) and processed using FlowJo Version 10.0.7 as previously described.^12^

### 4.10 Histology and Immunofluorescence

Scaffolds were fixed and prepared for immunofluorescence as previously described.^12,74^ Samples were imaged using a Leica SP5 confocal microscope using a 60× lens (N.A. 1.4) in the Wolfson Bioimaging facility, University of Bristol. Scaffolds were fixed in 4% paraformaldehyde before being paraffin embedded and sectioned using a Leica RM2125 Microtome into 10 μm sections floated onto polysine slides (VWR). Slides were first baked at 56 °C for approximately 5 h and then overnight at 37 °C. Slides were dewaxed by immersion into Histo-Clear (National Diagnostics) for 30–45 min before rehydration through a series of graded ethanol. Sections were then either stained for hematological stains or for immunofluorescence. Staining with May-Grünwald’s (VWR Chemicals) and Giemsa (Merck Millipore) according to manufacturer’s instructions was performed on rehydrated sections. For immunofluorescence, sections were washed once in PBS before blocking with PBS containing 4% BSA (PBSA) for 1 h. Slides were washed 5 times with PBS before incubating with primary antibody (primary and control antibodies are as stated in figure legends) for 1 h, again washed 5 times with PBS and incubated with Alexa 647 secondary antibody (Invitrogen) for 1 h. Slides were washed 5 times in PBS and incubated in DAPI to identify nuclei for 5 min before a further 2 washes. All steps were carried out at room temperature. For coverslips cells were fixed onto slide using 1% paraformaldehyde for 5 min before permeabilization in 0.05% Triton (in PBS) for 5 min at room temperature. Coverslips were then washed three times and blocked and stained with antibodies as above. Finally, all coverslips and slides were washed and mounted using mowiol (Calbiochem). Samples were imaged using a Leica SP5 confocal microscope using a 63× lens (N.A. 1.4) in the Wolfson Bioimaging facility, University of Bristol.

## Supplementary Material



## Notes

Additional FT-IR, NMR, Raman, and XPS spectra; table of XPS elemental composition analysis; images of polyHIPE scaffolds; and flow cytometry analysis of cell surface markers (PDF)
