# Supplementary material for: Hyaluronic Acid-Functionalized Highly Porous Polymeric Materials for Stem Cell Culture
Source: Chem Mater. 2025 Jul 22;37(15):5487–501. doi: 10.1021/acs.chemmater.5c00068 (PMC12355691; doi:10.1021/acs.chemmater.5c00068)
Supplement: Supplementary file 1 [file cm5c00068_si_001.pdf]

Supporting Information

Hyaluronic Acid-Functionalized Highly Porous Polymeric Materials for Stem Cell Culture

Joshua D. Swindell,[1] Despina Coursari,[1] Charlotte E. Severn,[2] Evelyn Calderon Espinosa,[1] Israa A. El-Shawaf,[3] David M. Haddleton,[1] Ashley M. Toye[2] and Ahmed M. Eissa*[1,4,5]

[1] Department of Chemistry, University of Warwick, Coventry. CV4 7AL, U.K.

[2] School of Biochemistry, Biomedical Sciences Building, University Walk, Bristol. BS8 1TD, U.K.

[3]Faculty of Engineering, Ain Shams University, Elsarayat St. 1, Abbaseya, Cairo 11517, Egypt

[4] Department of Polymers, Chemical Industries Research Division, National Research Centre, El Bohouth St. 33, Dokki, Giza 12622, Cairo, Egypt

[5] School of Pharmacy & Life Sciences and Research Institute of Healthcare Sciences, Faculty of Science and Engineering, University of Wolverhampton, Wolverhampton, WV1 1LY, U.K.

E-mail: A.M.Eissa@wlv.ac.uk

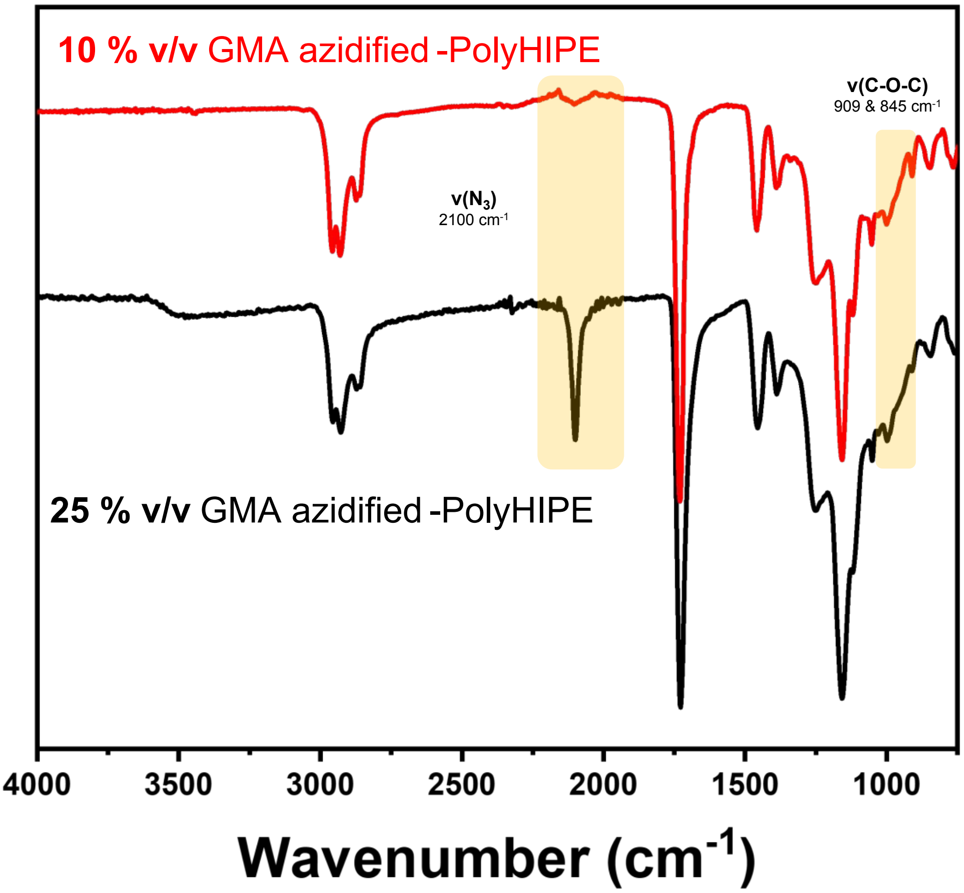

Figure 1S. Comparison of FT-IR spectra for 10 and 25 % v/v GMA azidified polyHIPEs.

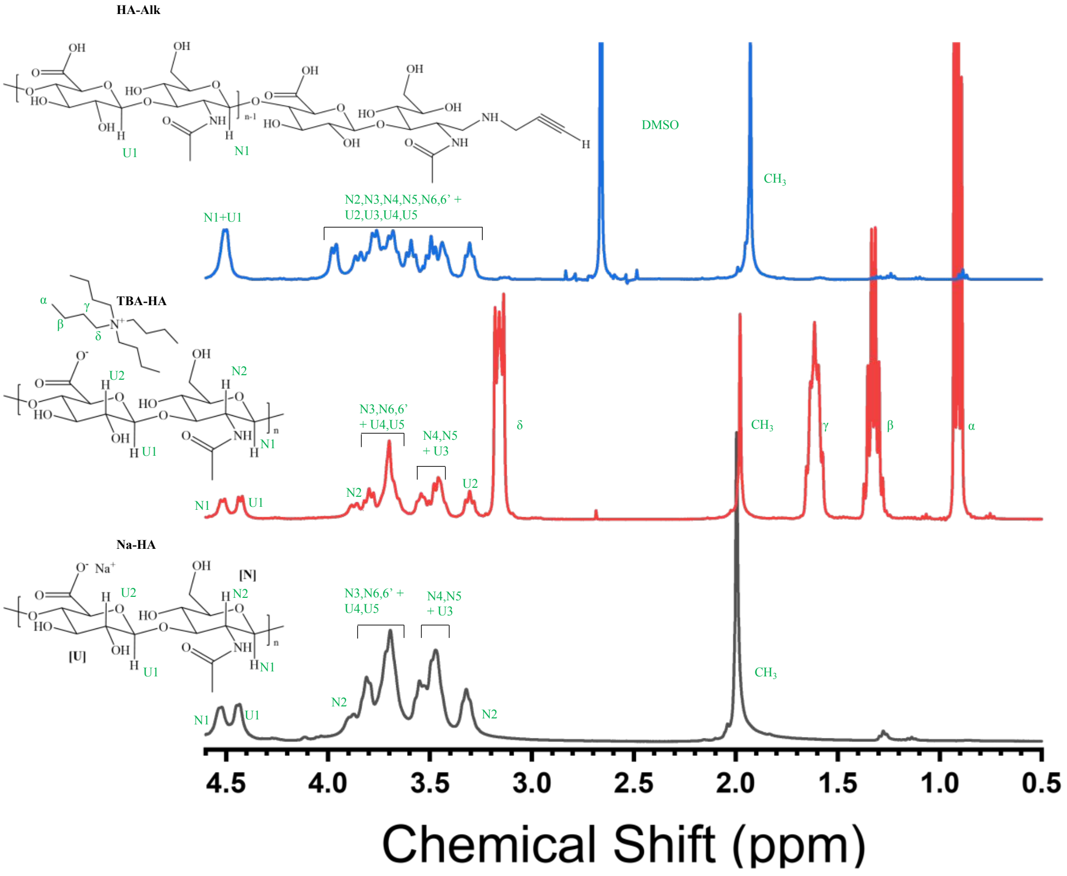

Figure 2S. ^{1}H NMR (D_2O, 400 MHz) spectra of Na-HA, TBA-HA and HA-Alk.

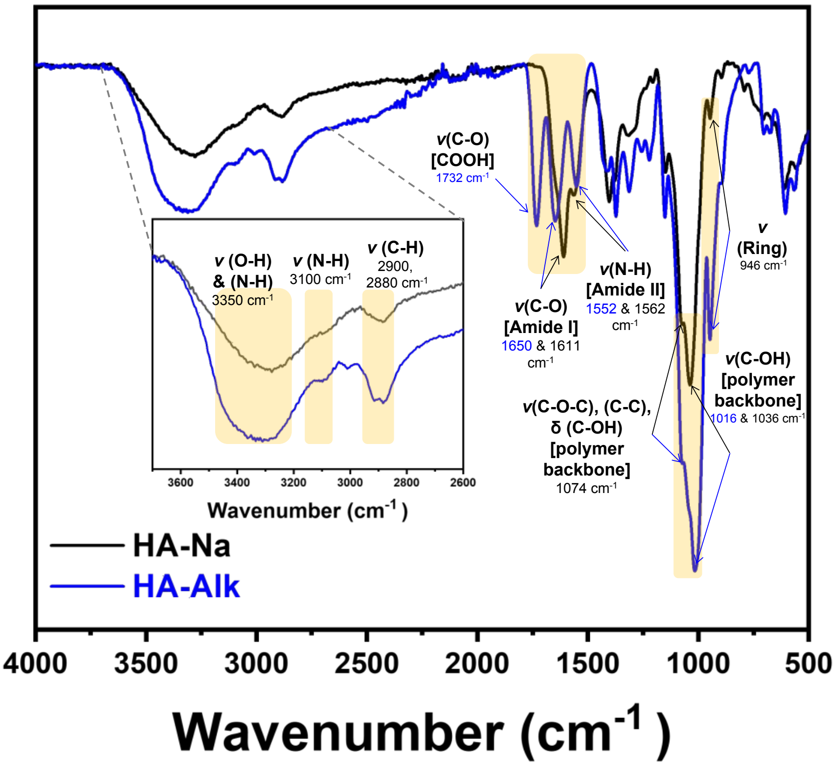

Figure 3S. **(A)** Overlaid FT-IR spectra of Na-HA and HA-Alk.

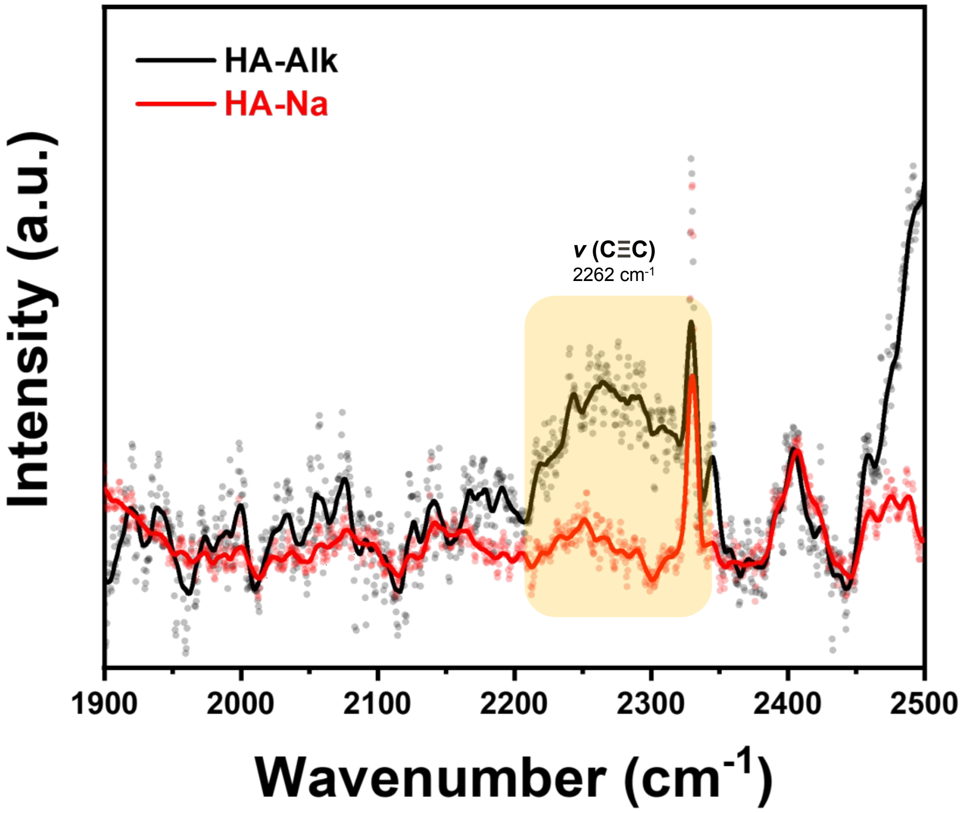

Figure 4S. Overlaid Raman spectra of Na-HA and HA-Alk showing appearance of C≡C stretch. Si 2p is suspected to be a contaminant.

Table 1S. XPS elemental composition analysis for HA and HA-Alk.

**Sample**	C 1s	O 1s	N 1s	Na 1s	Si 2p
Na-HA	53.33	35.46	2.88	3.56	4.78
HA-Alk	55.42	39.30	3.28	0	1.99

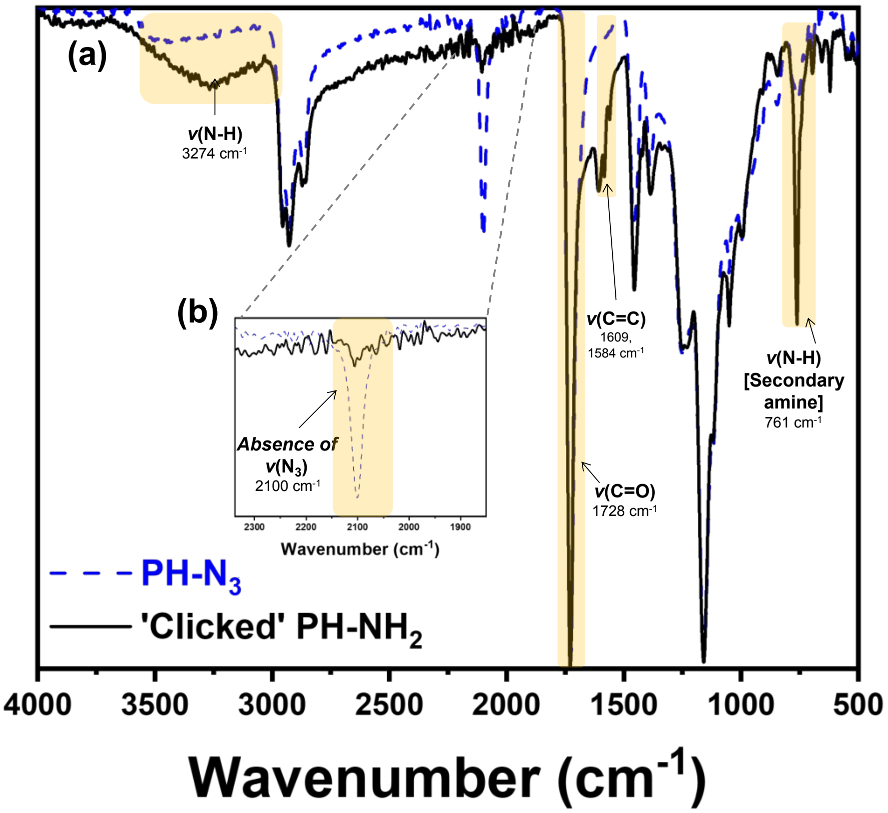

Figure 5S. (A) Overlaid FT-IR spectra of N_3-polyHIPE with clicked polyHIPE-NH_2 **(B)** Close up of azide peak reduction.

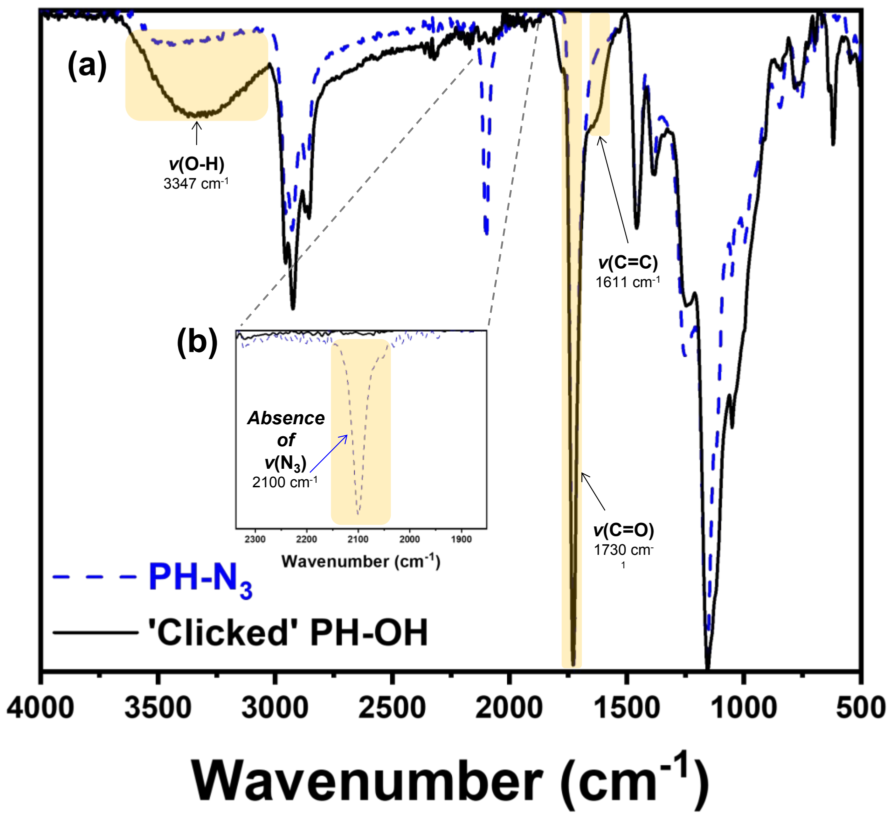

Figure 6S. (A) Overlaid FT-IR spectra of N_3-polyHIPE with clicked polyHIPE-OH **(B)** Close up of azide peak reduction.

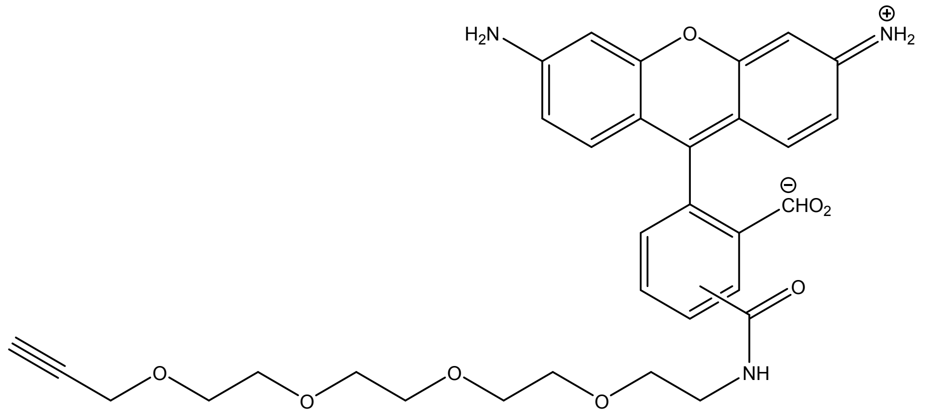

Figure 7S. Chemical structure of alkyne functionalised carboxyrhodamine-110

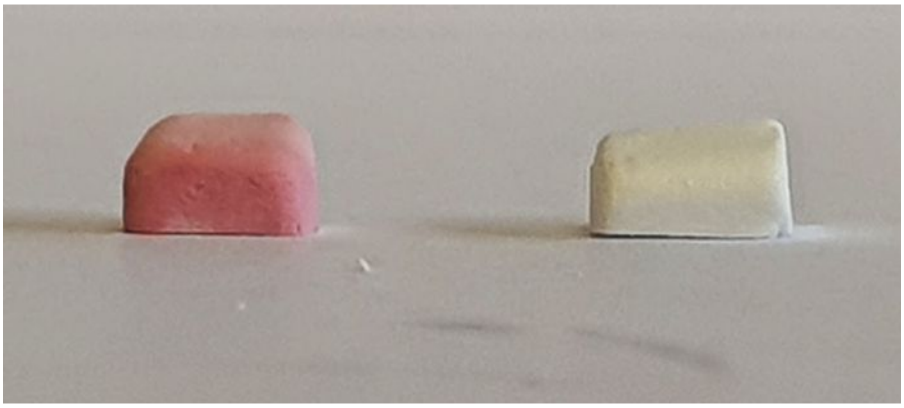

Figure 8S. Image of a polyHIPE scaffold clicked with carboxyrhodamine-110 (left) and unclicked N_3-polyHIPE scaffold as control (right).

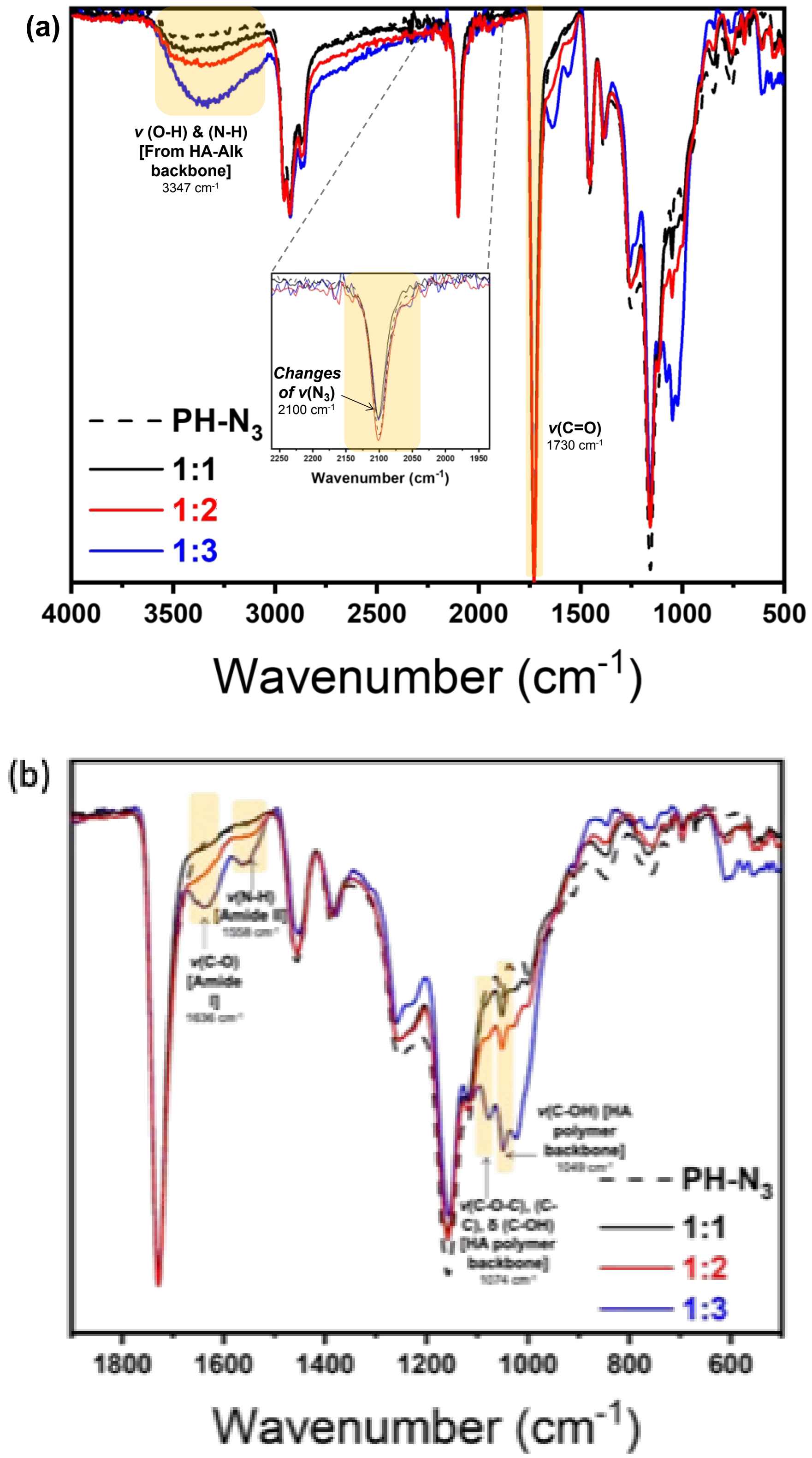

Figure 9S. (A) Overlaid FT-IR spectra of N_3-polyHIPE and HA clicked products at different HA-Alk to polyHIPE w/w ratios (1:1, 10mg:10mg; 1:2, 10mg:20mg; and 1:3, 10mg:30mg **(B)** close up of the region between 1800 cm^{-1} and 600 cm^{-1}

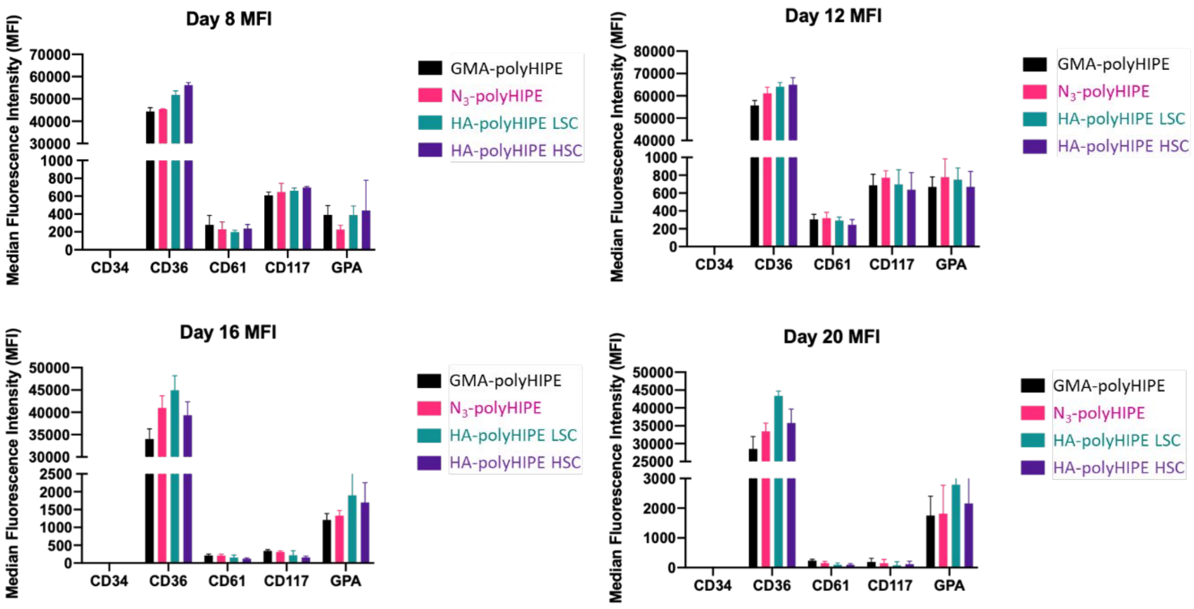

Figure 10S. Cell surface density of CD34, CD36, CD61, CD117 and GPA expressed as mean fluorescence intensity (MFI). Analysis of cell surface markers from polyHIPE scaffolds egress assessed every 4 days. Phenotype of scaffold egress in respect to the density of CD34, CD36, CD61, CD117 and GPA on the cell surface, MFI for each cell surface marker taken from the total live cell population. Analysis was conducted using the MACSQuant flow cytometer. N = 3 independent experiments in at least duplicate, error bars represent the standard error of the mean.
